# E-Cigarette and Cannabis Social Media Posts and Adolescent Substance Use

**DOI:** 10.1001/jamanetworkopen.2025.17611

**Published:** 2025-06-24

**Authors:** Julia Vassey, Junhan Cho, Erin A. Vogel, Trisha Iyer, Julia Chen-Sankey, Jennifer B. Unger

**Affiliations:** 1 Department of Population and Public Health Sciences, University of Southern California, Los Angeles; 2 TSET Health Promotion Research Center, University of Oklahoma Health Sciences Center, Oklahoma City; 3 Institute for Nicotine and Tobacco Studies, Rutgers University, New Brunswick, New Jersey; 4 School of Public Health, Rutgers University, Piscataway, New Jersey

## Abstract

**Importance:**

Adolescents are exposed to e-cigarette and cannabis content on social media. Understanding associations of these exposures with use and dual use of these products can guide regulations.

**Objective:**

To assess whether adolescent exposure to e-cigarette and/or cannabis content on social media, including posts by various content creators, is associated with e-cigarette, cannabis, and dual use.

**Design, Setting, and Participants:**

Two surveys, one longitudinal (study 1, baseline in 2021 to 2022) and one cross-sectional (study 2, fall 2023), were conducted among California high school students who completed questionnaires on computers in classrooms.

**Exposures:**

In study 1, the baseline was frequent exposure (weekly or more vs less frequent or none) to e-cigarette and/or cannabis social media posts. In study 2, the exposure (yes vs no) was to e-cigarette and/or cannabis posts from specific sources (friends, celebrities, microinfluencers, e-cigarette and/or cannabis brands, or unknown sources).

**Main Outcomes and Measures:**

For study 1, the primary outcome was solo e-cigarette, solo cannabis, or dual use initiation at 1-year follow-up among baseline never-users of e-cigarettes and cannabis. For study 2, the primary outcome was past-month use of e-cigarettes, cannabis, and dual use. Generalized estimating equations models adjusted for sociodemographic characteristics, mental health, other tobacco product use, social media use, and social environment.

**Results:**

In study 1, of 4232 adolescents (mean [SD] age, 17.0 [0.6] years; 2205 female [52.1%]), 968 (22.9%) reported frequent baseline exposure to e-cigarette posts and 507 (12.0%) reported exposure to cannabis posts on social media, broadly; 567 (13.4%) were frequently exposed to e-cigarette posts specifically on TikTok. Frequent exposure to cannabis social media posts was associated with solo e-cigarette use (adjusted odds ratio [AOR], 1.83; 95% CI, 1.11-3.01), solo cannabis use (AOR, 1.60; 95% CI, 1.07-2.38), and dual use (AOR, 1.71; 95% CI, 1.11-2.63) initiation at 1-year follow-up. Frequent exposure to e-cigarette posts on TikTok was associated with solo cannabis use (AOR, 1.74; 95% CI, 1.17-2.58) and dual use (e-cigarette and cannabis) initiation (AOR, 1.78; 95% CI, 1.19-2.66). In study 2, of 3380 adolescents (mean [SD] age, 17.0 [0.6] years; 1840 female [54.4%]), 195 (5.8%) were exposed to microinfluencer e-cigarette posts, and 152 (4.5%) were exposed to microinfluencer cannabis posts; 151 (4.5%) were exposed to friends’ e-cigarette posts, and 161 (4.8%) were exposed to friends’ cannabis posts. Exposure to e-cigarette (AOR, 2.67; 95% CI, 1.55-4.59) and cannabis (AOR, 2.14; 95% CI, 1.15-4.00) microinfluencer posts was associated with past-month cannabis use. Exposure to friends’ e-cigarette posts was associated with past-month dual use (AOR, 2.53; 95% CI, 1.24-5.19), whereas exposure to friends’ cannabis posts was associated with past-month cannabis use (AOR, 3.35; 95% CI, 1.94-5.78) and dual use (AOR, 2.46; 95% CI, 1.28-4.71).

**Conclusions and Relevance:**

In this survey study of California adolescents, exposure to e-cigarette or cannabis posts was associated with adolescent e-cigarette, cannabis, or dual use. Improvement of social media community guidelines and greater policy attention to co-use and marketing of e-cigarettes and cannabis may help prevent youth substance use.

## Key Points

**Question:**

Is adolescent exposure to e-cigarette and cannabis content on social media, including posts by various content creators, associated with e-cigarette, cannabis, and dual use of these substances?

**Findings:**

In this survey study of 7612 adolescents, frequent exposure to cannabis posts on social media broadly was associated with solo e-cigarette use, solo cannabis use, and dual use initiation a year later, whereas platform-specific exposure to e-cigarette posts on TikTok was associated with solo cannabis and dual use initiation. Exposure to e-cigarette and cannabis posts from microinfluencers was associated with past-month cannabis use, whereas exposure to e-cigarette posts from friends was associated with past-month dual use; exposure to friends’ cannabis posts was also associated with past-month cannabis and dual use.

**Meaning:**

Since exposure to e-cigarette or cannabis posts may contribute to adolescent e-cigarette, cannabis, or dual use, improvement of social media community guidelines and greater policy attention to co-use and marketing of e-cigarettes and cannabis may help prevent youth substance use.

## Teaser

This survey study examines associations of adolescent exposure to e-cigarette and cannabis content on social media with use of e-cigarettes and cannabis and dual use.

## Introduction

Nicotine e-cigarettes (hereafter, *e-cigarettes*) and cannabis products are among the most commonly used substances by adolescents in the US.^1,2^ In 2024, 3.5% of middle school and 7.8% of high school students reported current (past-month) e-cigarette use,^3^ whereas 8.3% of eighth graders and 29% of 12th graders reported using cannabis in the past year, 2023.^4^ Recreational cannabis legalization and the belief that cannabis use is not harmful may have contributed to increasing cannabis use among US adolescents over the past decade.^5,6^ Adolescents who use e-cigarettes risk progressing to using cannabis, and vice versa, and may concurrently use these products.^2,7,8,9^ E-cigarette and cannabis use each negatively impact adolescents’ brain development, lead to addiction, and are linked to depression and other mental health problems.^2,10,11,12^

Exposure to e-cigarette–related posts on social media is associated with adolescent use^13,14,15^ or intent to use e-cigarettes,^16^ and with adult e-cigarette and cannabis dual use.^17^ Exposure to cannabis content is associated with adolescent use or intent to use cannabis.^18,19^ However, the associations of adolescents’ exposure to e-cigarette and cannabis-related social media content, including platform-specific and source-specific exposure, with use of e-cigarettes, cannabis, or both substances, as well as cross-substance associations (eg, e-cigarette content with cannabis use), remain largely unknown. Understanding these associations is critical to informing social media policies and public health interventions to reduce adolescents’ nicotine and cannabis use.

Exposure to different social media platforms and e-cigarette or cannabis content sources may be associated differently with adolescent substance use behaviors. Prior research has found that daily TikTok^14^ and Instagram use^20^ and exposure to e-cigarette posts on those platforms were associated with e-cigarette use among adolescents, but the findings were inconsistent for Instagram.^14,20^ Exposure to cannabis marketing on Instagram was associated with cannabis use.^18^ Adolescents who reported exposure to e-cigarette posts from offline friends, online friends, and celebrities had greater odds of e-cigarette susceptibility than their unexposed peers.^21,22^ Young adult exposure to tobacco content from celebrities or influencers was associated with subsequent tobacco use initiation.^23^ Among adolescents and young adults who reported seeing e-cigarette–related content on social media, more than 40% reported seeing celebrity or influencer posts.^24^ Influencer marketing is particularly problematic since influencers may be perceived as more trusted sources than direct brand advertising, because of greater perceived authenticity and relatability of the influencer content.^25,26,27^ According to correspondent inference theory,^28^ consumers are more persuaded when they believe a spokesperson (eg, influencer) is intrinsically, not financially, motivated to promote a product. Microinfluencers (ie, noncelebrity influencers with approximately 10 000-100 000 followers) have promoted e-cigarettes alongside cannabis on social media,^29,30^ raising concerns for adolescent exposure to this potentially harmful content depicting both substances. The role of influencer marketing in adolescent e-cigarette, cannabis, and dual use patterns is unknown but is important to understand.

We conducted 2 studies among California adolescents to examine associations of exposure to e-cigarette and cannabis social media content with use of e-cigarettes, cannabis, or both. Study 1 used a longitudinal survey to assess the associations of frequent exposure to e-cigarette or cannabis social media posts with mutually exclusive outcomes: solo e-cigarette use, solo cannabis use, and dual use initiation at 1-year follow-up. On the basis of prior research,^13,14,15,16,17^ we hypothesized that frequent baseline exposure to e-cigarette posts would be associated with solo e-cigarette use, solo cannabis use, and dual use initiation a year later (hypothesis 1), and explored associations of exposure to cannabis posts with these substance use initiation outcomes (exploratory hypothesis). We also assessed the associations of platform-specific exposure to e-cigarette posts on TikTok, Instagram, and YouTube—platforms that are most popular among youth^31^ and are key sources of image-based and video-based user-generated and marketing tobacco-related content^30,32,33^—with solo e-cigarette, solo cannabis, and dual use initiation. On the basis of prior research,^14,20^ we hypothesized that frequent exposure to e-cigarette posts on TikTok at baseline, but not on Instagram or YouTube, would be associated with adolescent solo e-cigarette use, solo cannabis use, and dual use initiation at follow-up (hypothesis 2). Study 2, a cross-sectional survey, assessed associations of adolescent exposure to e-cigarette or cannabis user-generated posts (from friends), direct marketing (from e-cigarette or cannabis brands), indirect marketing (from microinfluencers or celebrities), or unknown sources with non–mutually exclusive outcomes: past-month e-cigarette, cannabis, or dual use. On the basis of prior research,^5,21,22,23,27,29^ we hypothesized that exposure to posts from friends, microinfluencers or celebrities, but not from brands or unknown sources, would be associated with past-month e-cigarette, cannabis, or dual use (hypothesis 3). We also explored the associations of exposure to cannabis posts from these sources with the same outcomes (exploratory hypothesis).

## Methods

### Participants and Procedures

For this survey study, participants in 10th and 11th grades from 24 socioeconomically and racially diverse high schools in Los Angeles, California, were recruited across 2 cohorts (eMethods in Supplement 1) in fall 2021 to spring 2022, with follow-up assessments in fall 2022 to spring 2023 for study 1^34,35^; 11th graders from 15 high schools were recruited out of 1 cohort in fall 2023 for study 2.^34^ Participants completed 30-minute REDCap surveys on Chromebook computers in classrooms. Parents provided written informed consent; participants provided written assent. This study followed the Strengthening the Reporting of Observational Studies in Epidemiology (STROBE) reporting guideline. The University of Southern California institutional review board approved all study procedures.

### Measures

#### Study 1

Solo e-cigarette use (any e-cigarette products for vaping nicotine), solo cannabis use (vaping, smoking, and edible cannabis products), and dual ever-use initiation of e-cigarettes and cannabis at 1-year follow-up were the primary outcomes (adapted from Population Assessment of Tobacco and Health [PATH] Study^36^) assessed among never-users of e-cigarettes and cannabis at baseline. E-cigarette products include any electronic vaping device with nicotine. Cannabis products include smoking cannabis, cannabis and THC (tetrahydrocannabinol) food or drinks, electronic devices to vape THC, cannabis, or hash oil. These dichotomized (yes or no) outcomes (eMethods in Supplement 1) were then combined in 1 multinomial variable with 4 mutually exclusive categories representing use initiation at 1-year follow-up: (1) solo e-cigarette use initiation (e-cigarette only); (2) solo cannabis use initiation (cannabis only); (3) dual use initiation (both substances); and (4) nonuse (noninitiation of either substances, reference). Study 1 independent variables (adapted from PATH^36^) were frequency of exposure to e-cigarette and cannabis posts (several times per day, daily, weekly, monthly or less, never, or don’t know). Responses were dichotomized to frequent exposure (at least weekly) vs all the other response choices combined (dichotomization was informed by prior research).^14,23^ For sensitivity analyses, responses were categorized more granularly (eMethods in Supplement 1). Exposure to e-cigarette posts was assessed both separately for Instagram, TikTok, and YouTube, as platform-specific exposure, and as a combined measure reflecting social media exposure broadly (on any of these platforms). Cannabis post exposure was only assessed broadly on social media because 1 of the 2 cohorts did not have platform-specific items for cannabis posts (eMethods in Supplement 1).

#### Study 2

Past-month e-cigarette, cannabis (vaping, smoking, and edible), and dual use of e-cigarettes and cannabis were the primary outcomes of study 2 assessed among both users and nonusers of e-cigarettes and/or cannabis. Survey items (adapted from PATH; eMethods in Supplement 1) measured days of e-cigarette and cannabis product use in the past 30 days. Owing to low cell counts for granular use (1-2 days, ≤3%; ≥3 days, ≤5% across substances), responses were dichotomized as any use (≥1 day) vs no use (0 days). Because of lower cell counts in study 2 (vs study 1), past-month e-cigarette, cannabis, and dual use were modeled as 3 separate, non–mutually exclusive outcomes rather than a single multinomial outcome. Ten independent variables represented ever-exposure (yes or no) to e-cigarette or cannabis posts from 5 sources: friends, celebrities, microinfluencers, e-cigarette and/or cannabis brands, and unknown. The source-specific exposure was assessed among those who reported at least weekly exposure to general e-cigarette and/or cannabis posts on social media; those with less frequent or no general exposure (who were not assessed for source-specific exposure) remained in the analysis and were grouped with those who reported not seeing source-specific posts (eMethods in Supplement 1).

### Statistical Analysis

For study 1, a generalized estimating equation multivariable model for multinomial logistic regression, accounting for school-level clustering, was used to assess associations of frequent baseline exposure (at least weekly vs less frequent or none) to e-cigarette (hypothesis 1) and cannabis (exploratory) social media posts, and platform-specific frequent baseline exposure to e-cigarette posts on TikTok, Instagram, and YouTube (hypothesis 2), with solo e-cigarette use, solo cannabis use, and dual use initiation at 1-year follow-up among baseline e-cigarette and cannabis never-users. We also conducted supplementary sensitivity analyses using more granular frequency of exposure levels (eMethods in Supplement 1). For study 2, generalized estimating equation multivariable models for binomial logistic regression were used to assess the associations of source-specific exposures to e-cigarette (hypothesis 3) and cannabis (exploratory hypothesis) social media posts with past-month e-cigarette, cannabis, and dual use.

In both studies, all multivariable regression models were adjusted for self-reported sociodemographic characteristics (age, sex assigned at birth, sexual identity, race and ethnicity, parental education, and family income^37^), mental health representing internalizing disorders (generalized anxiety disorder and social phobia),^38^ other tobacco product use (combustible cigarettes, tobacco heating sticks, or other heated tobacco devices, oral nicotine products, big cigars, little cigars and cigarillos, and hookah), social media use, social environment (number of friends who used e-cigarettes or cannabis), and school clustering (eMethods in Supplement 1). The choice of covariates was informed by studies that found that the associations of exposure to tobacco social media content with e-cigarette or cannabis use differed according to these covariates.^14,20,23,39,40^

In both studies, adjusted odds ratios (AORs) with 95% CIs were reported with statistical significance set at *P* < .05 (2-tailed), using Benjamini-Hochberg correction to control false discovery rate at 0.05. Missing data on the independent variables and covariates (nonresponse and prefer not to respond answers) were retained (eTables 1-8 in Supplement 1). A negligible number of missing observations in key outcomes—9 for past-month e-cigarette use, 14 for past-month cannabis use, and 10 for past-month dual use (all <1%)—were handled using listwise deletion. All statistical analyses were conducted using R statistical software version 4.2.2 (R Project for Statistical Computing) and SAS statistical software version 9.4 (SAS Institute).

## Results

### Study 1

For study 1 (longitudinal), 5206 students completed both baseline and follow-up surveys (88% retention rate; eFigure and eMethods in Supplement 1). The analytic sample consisted of 4232 (81%) baseline never-users of e-cigarettes and cannabis (mean [SD] age, 17.0 [0.6] years at follow-up; 2205 female [52.1%]). At follow-up, 153 participants (3.6%) reported solo e-cigarette use initiation, 166 (3.9%) reported solo cannabis use initiation, and 190 (4.5%) reported dual use initiation (e-cigarette and cannabis); 968 (22.9%) reported frequent (at least weekly) baseline exposure to e-cigarette–related social media content, and 507 (12.0%) reported frequent baseline exposure to cannabis-related social media content. In addition, 567 participants (13.4%) reported frequent (at least weekly) baseline exposure to e-cigarette-related content on TikTok, 590 (13.9%) reported exposure on Instagram, and 471 (11.1%) reported exposure on YouTube (Table 1). Bivariate associations are reported in eTables 2 to 4 in Supplement 1.

**Table 1.** Participant Characteristics in Study 1

Characteristics	Participants, No. (%) (N = 4232)
Age, mean (SD), y	17 (0.57)
Sex assigned at birth	
Male	1904 (45.0)
Female	2205 (52.1)
Sexual orientation, heterosexual	3201 (75.6)
Race	
Asian	1476 (34.9)
White	761 (18.0)
Multiple race	831 (19.6)
Other^a^	878 (20.7)
Ethnicity, Hispanic	1994 (47.1)
Social media use (daily or multiple times per day)^b^	3639 (86.0)
Exposure to substance-related content on social media (weekly, daily, or multiple times per day)^b^	
E-cigarettes	968 (22.9)
Cannabis	507 (12.0)
Exposure to e-cigarette–related platform-specific content (weekly, daily, or multiple times per day)	
TikTok	567 (13.4)
Instagram	590 (13.9)
YouTube	471 (11.1)
Substance ever-use initiation as follow-up	
Solo e-cigarette use^c^	153 (3.6)
Solo cannabis use^d^	166 (3.9)
Dual use of e-cigarettes and cannabis	190 (4.5)

^a^ Other includes African American or Black, American Indian or Alaska Native, Native Hawaiian and Pacific Islander, and unspecified other self-reported race categories.

^b^ Social media include Instagram, TikTok, and YouTube.

^c^ Refers to any electronic cigarette for vaping nicotine.

^d^ Refers to smoking cannabis (pot, weed, hash, reefer, bud, or grass), cannabis or THC (tetrahydrocannabinol) foods or drinks (pot brownies, edibles, cookies, cakes, butter, or oil), electronic device to vape THC or hash oil (liquid pot, cannabis oil, weed pen, or vape pods).

Hypothesis 1 was not supported: there was no association between baseline frequent exposure to e-cigarette content with solo e-cigarette use, solo cannabis use (after adjusting for multiple testing), or dual use initiation at follow-up (Table 2). However, frequent exposure to cannabis content (exploratory hypothesis) was associated with solo e-cigarette use (AOR, 1.83; 95% CI, 1.11-3.01), solo cannabis use (AOR, 1.60; 95% CI, 1.07-2.38), and dual use (AOR, 1.71; 95% CI, 1.11-2.63) initiation (Table 2).

**Table 2.** Multinomial Logistic Regression Analysis of Exposure to E-Cigarette and Cannabis Posts and Substance Use Initiation (Study 1)^a^

Independent variables	Outcomes (mutually exclusive)						Model statistics			
	Solo e-cigarette ever-use initiation (n = 153)		Solo cannabis ever-use initiation (n = 166)		Dual ever-use (e-cigarettes and cannabis) initiation (n = 190)		2-Log likelihood		χ^2^_60_	Nagelkerke *R*^2^
	AOR (95% CI)	*P* value	AOR (95% CI)	*P* value	AOR (95% CI)	*P* value	Intercept only	Full model		
Model 1: Frequent (at least weekly) exposure to e-cigarette posts on social media^b^	1.30 (0.85-1.99)	.23	1.45 (1.02-2.06)	.12	1.37 (0.94-2.01)	.15	4224.54	3181.44	1043.10	0.25
Model 2: Frequent (at least weekly) exposure to cannabis posts on social media^b^^,^^c^	1.83 (1.11-3.01)	.03	1.60 (1.07-2.38)	.03	1.71 (1.11-2.63)	.047	4224.54	3175.72	1048.83	0.25

Abbreviation: AOR, adjusted odds ratio.

^a^ Details on models and covariates are shown in the Methods section. The reference category for independent variables was exposure monthly or less, never, uncertain and unreported, all combined. The reference category for each of the 3 mutually exclusive outcome categories was nonusers of e-cigarettes and cannabis at follow-up (n = 3727).

^b^ Refers to exposure to e-cigarette content on at least 1 of the 3 social media platforms: Instagram, TikTok, and YouTube.

^c^ Exposure to cannabis content on social media was assessed as exposure on social media in general in cohort 1 and as exposure on at least 1 of the 3 social media platforms: Instagram, TikTok, and YouTube in cohort 2. As part of harmonization, responses from cohort 2 were combined into a single variable (exposure on any of the 3 platforms [TikTok, Instagram, and YouTube]) to match cohort 1 (details are described in eMethods in Supplement 1).

Hypothesis 2 was partially supported. Frequent exposure to e-cigarette posts on TikTok was associated with solo cannabis use initiation (AOR, 1.74; 95% CI, 1.17-2.58) and dual use initiation (AOR, 1.78; 95% CI, 1.19-2.66), but not solo e-cigarette use initiation (Table 3).

**Table 3.** Multinomial Logistic Regression Analysis of Platform-Specific Exposure to E-Cigarette Posts and Substance Use Initiation (Study 1)^a^

Independent variables	Outcomes (mutually exclusive)						Model statistics			
	Use initiation solo e-cigarette (n = 153)		Use initiation solo cannabis (n = 166)		Use initiation dual (e-cigarettes and cannabis) (n = 190)		2-Log likelihood		χ^2^_60_	Nagelkerke *R*^2^
	AOR (95% CI)	*P* value	AOR (95% CI)	*P* value	AOR (95% CI)	*P* value	Intercept only	Full model		
Model 1: Frequent (at least weekly) exposure to e-cigarette posts on TikTok	1.50 (0.93-2.42)	.10	1.74 (1.17-2.58)	.009	1.78 (1.19-2.66)	.02	4224.54	3174.75	1049.79	0.25
Model 2: Frequent (at least weekly) exposure to e-cigarette posts on Instagram	1.02 (0.60-1.73)	>.99	1.47 (0.99-2.18)	.18	1.08 (0.69-1.71)	>.99	4224.54	3184.69	1039.85	0.25
Model 3: Frequent (at least weekly) exposure to e-cigarette posts on YouTube	1.09 (0.61-1.94)	>.99	1.09 (0.67-1.76)	>.99	0.87 (0.51-1.50)	>.99	4224.54	3187.58	1036.96	0.25

Abbreviation: AOR, adjusted odds ratio.

^a^ Details on models and covariates are shown in the Methods section. The reference category for independent variables is exposure monthly or less, never, uncertain and unreported, all combined. The reference category for each of the 3 mutually exclusive outcome categories is nonusers of e-cigarettes and cannabis at follow-up (n = 3727).

Results of the sensitivity analyses for frequency of exposure to e-cigarette and cannabis posts (eTables 5 and 6 in Supplement 1) remained largely consistent with those in the main analyses, except that frequent exposure to e-cigarette posts on TikTok (vs no exposure) was associated with solo e-cigarette use initiation, whereas frequent exposure to cannabis posts on social media was not associated with solo e-cigarette use initiation.

### Study 2

For study 2 (cross-sectional), 3380 adolescents (77%) completed the survey (eFigure in Supplement 1). Of the 3380 participants (mean [SD] age, 17.0 [0.6] years; 1840 female [54.4%]), 170 (5.0%) reported using e-cigarettes, 265 (7.8%) reported using cannabis, and 116 (3.4%) reported dual use in the past month. (Substance use categories were not mutually exclusive.) The most prevalent source of e-cigarette content was unknown (611 participants [18.1%]), followed by e-cigarette brands (407 participants [12.0%]), microinfluencers (195 participants [5.8%]), friends (151 participants [4.5%]), and celebrities (131 participants [3.9%]). The most prevalent source of cannabis content was also unknown (353 participants [10.4%]), followed by cannabis brands (243 participants [7.2%]), friends (161 participants [4.8%]), microinfluencers (152 participants [4.5%]), and celebrities (108 participants [3.2%]) (Table 4). Bivariate associations are reported in eTables 7 and 8 in Supplement 1.

**Table 4.** Participant Characteristics in Study 2

Characteristics	Participants, No. (%) (N = 3380)
Age, mean (SD), y	17 (0.61)
Sex assigned at birth	
Male	1493 (44.2)
Female	1840 (54.4)
Sexual orientation, heterosexual	2402 (71.1)
Race	
Asian	1295 (38.3)
White	640 (18.9)
Multiple race	625 (18.5)
Other^a^	715 (21.1)
Ethnicity, Hispanic	1638 (48.5)
Social media use (daily or multiple times per day)^b^	2873 (85.0)
Exposure to source-specific e-cigarette content on social media^c^	
Posts from friends	151 (4.5)
Posts from microinfluencers	195 (5.8)
Posts from celebrities	131 (3.9)
Posts from e-cigarette brands	407 (12.0)
Posts from unknown sources	611 (18.1)
Exposure to source-specific cannabis content on social media^c^	
Posts from friends	161 (4.8)
Posts from microinfluencers	152 (4.5)
Posts from celebrities	108 (3.2)
Posts from cannabis brands	243 (7.2)
Posts from unknown sources	353 (10.4)
Past 30-d substance use	
E-cigarettes^d^	170 (5.0)
Cannabis^e^	265 (7.8)
Dual use of e-cigarettes and cannabis	116 (3.4)

^a^ Other includes African American or Black, American Indian or Alaska Native, Native Hawaiian and Pacific Islander, and unspecified other self-reported race categories.

^b^ Social media include Instagram, TikTok, and YouTube.

^c^ Source-specific exposure to e-cigarette and cannabis content was assessed among participants who reported at least weekly exposure to e-cigarette or cannabis content on social media.

^d^ Refers to any electronic cigarette for vaping nicotine.

^e^ Refers to smoking cannabis (pot, weed, hash, reefer, bud, or grass), cannabis or THC (tetrahydrocannabinol) foods or drinks (pot brownies, edibles, cookies, cakes, butter, oil), electronic device to vape THC or hash oil (liquid pot, cannabis oil, weed pen, and vape pods).

Hypothesis 3 was partially supported: exposure to e-cigarette posts from friends was associated with past-month dual use of e-cigarettes and cannabis (AOR, 2.53; 95% CI, 1.24-5.19). Exposure to e-cigarette posts from microinfluencers was associated with past-month cannabis use (AOR, 2.67; 95% CI, 1.55-4.59) (Table 5). Exposure to cannabis posts (exploratory hypothesis) from friends was associated with past-month cannabis (AOR, 3.35; 95% CI, 1.94-5.78) and dual use (AOR, 2.46; 95% CI, 1.28-4.71). Exposure to cannabis posts from microinfluencers was associated with past-month cannabis use (AOR, 2.14; 95% CI, 1.15-4.00) (Table 5).

**Table 5.** Logistic Regression Analysis of Source-Specific Exposure to E-Cigarette and Cannabis Posts and Past-Month Substance Use (Study 2)^a^

Variables	E-cigarette posts						Cannabis posts					
	Model 1		Model 2		Model 3		Model 4		Model 5		Model 6	
	E-cigarette use (n = 170)^b^		Cannabis use (n = 265)^c^		E-cigarettes and cannabis dual-use (n = 116)^d^		E-cigarette use (n = 170)^b^		Cannabis use (n = 265)^c^		E-cigarettes and cannabis dual-use (n = 116)^d^	
	AOR (95% CI)	*P* value	AOR (95% CI)	*P* value	AOR (95% CI)	*P* value	AOR (95% CI)	*P* value	AOR (95% CI)	*P* value	AOR (95% CI)	*P* value
Source of posts												
Friends	2.22 (1.08-4.57)	.15	1.41 (0.81-2.45)	.57	2.53 (1.24-5.19)	.04	1.54 (0.77-3.10)	>.99	3.35 (1.94-5.78)	<.001	2.46 (1.28-4.71)	.03
Microinfluencers	1.04 (0.52-2.06)	.92	2.67 (1.55-4.59)	.002	2.18 (1.07-4.42)	.08	0.99 (0.45-2.19)	.99	2.14 (1.15-4.00)	.04	1.94 (0.91-4.15)	.21
Celebrity influencers	0.84 (0.34-2.10)	>.99	0.81 (0.40-1.63)	.55	0.96 (0.36-2.58)	>.99	0.73 (0.28-1.89)	.87	0.60 (0.25-1.45)	.32	0.49 (0.20-1.22)	.20
Brands	1.65 (0.98-2.05)	.16	0.86 (0.54-1.38)	.67	1.74 (1.01-3.01)	.08	0.76 (0.36-1.58)	.67	1.19 (0.68-2.06)	.54	1.31 (0.66-2.60)	.56
Unknown	1.12 (0.61-2.05)	.91	0.86 (0.55-1.36)	.88	1.00 (0.53-1.88)	.99	0.81 (0.42-1.56)	>.99	1.42 (0.88-2.28)	.24	1.20 (0.63-2.28)	.58
Model statistics												
2-Log likelihood												
Intercept only	1328.33		1845.19		1009.59		1328.33		1845.19		1009.59	
Full model	728.61		1150.45		647.15		734.07		1133.58		655.43	
χ^2^_60_	599.72		694.74		362.43		594.26		711.60		354.15	
Nagelkerke *R*^2^	0.45		0.38		0.36		0.45		0.39		0.35	

Abbreviation: AOR, adjusted odds ratio.

^a^ Details on models and covariates are shown in the Methods section. The reference group for independent variables (no exposure) included participants who explicitly reported no exposure to e-cigarette and/or cannabis content from specific sources and those who reported less than weekly or no exposure to non–source-specific e-cigarette and/or cannabis posts and were therefore not shown the source-specific exposure question, but remained in the analysis as part of the unexposed group.

^b^ Reference for e-cigarette use outcome is never use or no past-month use of e-cigarettes.

^c^ Reference for cannabis use outcome is never use or no past-month use of cannabis.

^d^ Reference for dual-use outcome is never use or no past-month use of e-cigarette and/or cannabis.

## Discussion

Using longitudinal and cross-sectional surveys of high school adolescents in California, this study examined the associations of frequent exposure to e-cigarette and cannabis social media posts, and from specific content creators, with subsequent initiation and past-month use and dual use of e-cigarettes and cannabis. The studies found that although baseline frequent exposure to e-cigarette content broadly on social media was not associated with subsequent e-cigarette or cannabis use initiation, frequent exposure to cannabis content was associated with solo e-cigarette use (inconsistently), solo cannabis use, and dual use initiation 1 year later. Frequent exposure to e-cigarette posts on TikTok and exposure to e-cigarette and/or cannabis social media posts from friends and microinfluencers were associated with cannabis and dual use initiation or past-month use.

The study findings suggest that exposure to e-cigarette or cannabis content on social media is, overall, a weak or inconsistent factor associated with e-cigarette use among adolescents, aligning with prior research among adults.^17^ Frequent exposure to e-cigarette content broadly on social media was not associated with solo e-cigarette use initiation, which was contrary to our hypothesis. Frequent exposure to cannabis posts was inconsistently associated with solo e-cigarette use initiation (significant in the main analysis but not in the sensitivity analysis). Similarly, platform-specific exposure to e-cigarette posts on TikTok was inconsistently associated with solo e-cigarette use initiation (not significant in the main analysis but significant in the sensitivity analysis). Finally, no source-specific exposure to e-cigarette or cannabis posts was significantly associated with past-month e-cigarette use. These findings may reflect adolescents’ greater awareness of e-cigarette health risks (eg, through educational campaigns among youth)^41,42^ and to the declining trend in e-cigarette use among adolescents nationwide,^3^ which could also contribute to reduced youth appeal of e-cigarette posts. The findings may also stem from fewer adolescent solo e-cigarette initiators than solo cannabis and dual use initiators, and fewer past-month e-cigarette users than cannabis users, reducing the statistical power to detect significant associations for solo e-cigarette initiation and past-month e-cigarette use. For cannabis use, exposure to e-cigarette and cannabis posts showed not only significant associations with corresponding substance use (ie, cannabis content with cannabis use), but also cross-substance associations (ie, e-cigarette content with cannabis use). These associations could potentially be explained by low-risk perceptions of recreationally legalized cannabis,^43^ misperceptions about the product being promoted due to visual similarity between disposable nicotine and cannabis vape devices,^29^ and lack of stringent online sales policies restricting access to cannabis products for minors.^5,6,44^ Significant findings for dual use of e-cigarettes and cannabis may suggest that cannabis use fuels dual use, potentially to enhance psychoactive effects, offset cannabis’ adverse effects with nicotine, or due to behavioral cues in social settings where multiple substances are used.^45^

In support of our hypotheses, exposure to e-cigarette posts on TikTok was associated with solo e-cigarette use (but inconsistently), solo cannabis use, and dual use initiation. No associations were found for YouTube, whereas exposure to e-cigarette posts on Instagram was significantly associated with cannabis use initiation in the sensitivity analysis before, but not after, adjustment for multiple testing. These differences may reflect platform-specific engagement. TikTok’s interactive features, such as trending challenges and viral hashtags, encourage user participation and content visibility.^46^ In addition, youth report greater exposure to pronicotine or tobacco content on TikTok than on other platforms,^24^ which may contribute to its greater associations.

Prevalence of self-reported exposure to a specific source of e-cigarette or cannabis content in study 2 was lower for user-generated (from friends) and indirect marketing content (from celebrities and microinfluencers), with more participants reporting exposure to direct marketing from e-cigarette and cannabis brands, and even more participants not recalling who posted e-cigarette and cannabis content, to which they were exposed. Although more adolescents reported exposure from an unknown source, it was not associated with substance use, suggesting it may have been less salient than more memorable content from specific sources. In partial support of our hypothesis, exposure to e-cigarette posts from friends was associated with past-month dual use, but not e-cigarette use, after adjusting for multiple testing, despite friends’ influence being an established factor associated with adolescent e-cigarette use.^21,23^ Exposure to cannabis posts from friends was associated with cannabis use and also associated with dual use, highlighting the role of adolescents’ social environment in these substance use behaviors. Prior observational studies found associations between exposure to e-cigarette content posted by celebrities or influencers (assessed as a combined measure) with susceptibility to^22^ and initiation of^23^ e-cigarette use among adolescents and young adults.^22,23^ Adding to the existing literature, this study examined associations of exposure to celebrity and influencer content separately. Exposure to e-cigarette and cannabis posts from microinfluencers, but not celebrities, was associated with past-month cannabis use. This finding suggests that influencer marketing of e-cigarettes and cannabis could contribute to the risk of cannabis use. For adolescents who perceive cannabis as low risk, e-cigarette promotion alongside cannabis may further reduce the perceived risk of e-cigarettes and encourage dual use, especially when nicotine and cannabis vape devices look similar, as is often the case in today’s marketplace, creating misperceptions about which product is being promoted.^29^ Among marketing sources, exposure to microinfluencer e-cigarette and cannabis posts was the only significant variable (ie, of past-month cannabis use), possibly due to influencers’ perceived authenticity and relatability,^25,26,27^ unlike brands or celebrities. Although influencers receive brand sponsorships, partnerships often stem from their product knowledge and interest.^47^ Microinfluencers are also cost-effective,^48^ making them attractive for e-cigarette and cannabis brand partnerships.^29,49^

This study emphasizes the need for improved enforcement of social media community guidelines, especially on TikTok, regulation of social media substance-related marketing, particularly influencer marketing, and greater research and policy attention to marketing of e-cigarette and cannabis products. Cannabis promotion is exacerbated by the lack of federal regulations in the US restricting cannabis marketing and online sales to youth.^50^ Although more than 20 states,^51^ including California,^52^ have adopted laws prohibiting youth-targeted ads, enforcement remains challenging, especially on social media, where ambiguous regulations allow marketing sources to bypass restrictions.^18,29,53^ Although states where cannabis is not legalized have stricter cannabis marketing laws,^51^ adolescents in any state remain vulnerable to cannabis-related social media content owing to the borderless nature of social media, where exposure may not be limited by local policy. Social media platforms regulate substance-related content differently. Most platforms prohibit paid tobacco ads,^54^ with Facebook, Instagram, and TikTok also prohibiting influencer tobacco marketing.^54^ Some platforms restrict cannabis promotion,^55,56^ whereas Facebook, Instagram, and YouTube have eased cannabis marketing restrictions.^57,58^ User-generated content is largely unregulated, aside from limited platform-specific restrictions (eg, posting substance-related content featuring young people).^54,59,60^ Despite these policies, violations persist.^30,33,60,61^ Platforms could leverage artificial intelligence to detect and remove substance-related posts.^33,62,63^ Exposure to user-generated and marketing substance-related content, regardless of a specific product, may encourage adolescent experimentation with different substances, highlighting the need for improving social media community guidelines and regulations as a potential strategy to mitigate public health risks associated with youth e-cigarette and cannabis use.

### Limitations

This study has limitations that should be mentioned. Since the California-based cohorts in this study did not use probability-based sampling or apply sampling weights, the findings may not generalize to all US adolescents, although the study samples reflect the projected future US ethnic diversity.^64^ In addition, since participating schools are located in the demographically diverse Inland Empire and Greater Los Angeles counties (eMethods in Supplement 1), the study samples are comparable to Los Angeles County population. Self-reported survey responses might be prone to social desirability (eg, underreporting substance use despite assurances of confidentiality) and recall bias (eg, accurately recalling exposure sources). Owing to differences in cannabis exposure assessment across survey cohorts, study 1 could not assess platform-specific cannabis exposure. Analysis in study 2 was cross-sectional, precluding causal inference or trend analysis. A smaller number of solo e-cigarette users compared with cannabis and dual users (study 1), limited exposure to posts from friends, celebrities, and microinfluencers, along with fewer past-month e-cigarette and dual users than cannabis users (study 2) may have reduced statistical power to detect additional significant associations. Although the dichotomization of the past-30-day use outcomes in study 2 limits interpretation of use frequency, prior meta-analyses^13,65^ have shown associations between various variables (eg, social media exposure) and dichotomized past-30-day tobacco use (≥1 day vs 0 days), supporting its clinical relevance.

## Conclusions

Exposure to e-cigarette content on TikTok and cannabis content broadly on social media was associated with solo cannabis and dual use initiation but showed inconsistent associations with solo e-cigarette initiation. Similarly, exposure to e-cigarette and cannabis posts from friends or marketing sources (ie, brands, celebrities, and microinfluencers) was not associated with past-month e-cigarette use, whereas exposure to posts from friends and microinfluencers was associated with past-month cannabis or dual use. These findings suggest that exposure to e-cigarette and cannabis content on social media has a more consistent association with cannabis use and dual use than with solo e-cigarette use. Future research should explore whether shifts in tobacco and cannabis policies (eg, state-level cannabis legalization or a tightened premarket application review process for e-cigarette product market entry), social media community guideline enforcement (eg, prompt detection and removal of influencer vaping posts, regardless of the substance depicted), and prevention programs contribute to these associations. Improving social media guidelines and regulation of substance-related marketing, especially influencer promotions of e-cigarettes and cannabis, may help limit adolescent exposure and mitigate public health risks associated with youth e-cigarette and cannabis use.

## Notes

Supplement 1. **eFigure**. Consortium diagrams for study 1 and study 2 **eTable 1.** Survey participation and retention rates across two study waves (study 1) **eTable 2.** Bivariate associations of exposure to e-cigarette and cannabis posts with substance use initiation (study 1) **eTable 3.** Bivariate associations of platform-specific exposure to e-cigarette posts with substance use initiation (study 1) **eTable 4.** Bivariate associations of covariates with substance use initiation (study 1) **eTable 5.** Multinomial logistic regression analysis of exposure to e-cigarette and cannabis posts and substance use initiation (study 1) **eTable 6.** Multinomial logistic regression analysis of platform-specific exposure to e-cigarette posts and substance use initiation (study 1) **eTable 7.** Bivariate associations of source-specific exposure to e-cigarette and cannabis posts with past-month substance use (study 2) **eTable 8.** Bivariate associations of covariates with past-month substance use (study 2) **eMethods.** Additional details on data sources, participants, survey measures, and analysis
eReferences

Supplement 2.
Data Sharing Statement
