# Supplementary material for: E-Cigarette and Cannabis Social Media Posts and Adolescent Substance Use
Source: JAMA Netw Open. 2025 Jun 24;8(6):e2517611. doi: 10.1001/jamanetworkopen.2025.17611 (PMC12188343; doi:10.1001/jamanetworkopen.2025.17611)
Supplement: Supplement 1. — eFigure. Consortium diagrams for study 1 and study 2 eTable 1. Survey participation and retention rates across two study waves (study 1) eTable 2. Bivariate associations of exposure to e-cigarette and cannabis posts with substance use initiation (study 1) eTable 3. Bivariate associations of platform-specific exposure to e-cigarette posts with substance use initiation (study 1) eTable 4. Bivariate associations of covariates with substance use initiation (study 1) eTable 5. Multinomial logistic regression analysis of exposure to e-cigarette and cannabis posts and substance use initiation (study 1) eTable 6. Multinomial logistic regression analysis of platform-specific exposure to e-cigarette posts and substance use initiation (study 1) eTable 7. Bivariate associations of source-specific exposure to e-cigarette and cannabis posts with past-month substance use (study 2) eTable 8. Bivariate associations of covariates with past-month substance use (study 2) eMethods. Additional details on data sources, participants, survey measures, and analysis eReferences [file jamanetwopen-e2517611-s001.pdf]

Supplemental Online Content

Vassey J, Cho J, Vogel EA, Iyer T, Chen-Sankey J, Unger JB. E-cigarette and cannabis social media posts and adolescent substance use. *JAMA Netw Open*. 2025;8(6):e2517611. doi:10.1001/jamanetworkopen.2025.17611

eFigure. Consortium diagrams for study 1 and study 2
eTable 1. Survey participation and retention rates across two study waves (study 1)
eTable 2. Bivariate associations of exposure to e-cigarette and cannabis posts with substance use initiation (study 1)
eTable 3. Bivariate associations of platform-specific exposure to e-cigarette posts with substance use initiation (study 1)
eTable 4. Bivariate associations of covariates with substance use initiation (study 1)
eTable 5. Multinomial logistic regression analysis of exposure to e-cigarette and cannabis posts and substance use initiation (study 1)
eTable 6. Multinomial logistic regression analysis of platform-specific exposure to e-cigarette posts and substance use initiation (study 1)
eTable 7. Bivariate associations of source-specific exposure to e-cigarette and cannabis posts with past-month substance use (study 2)
eTable 8. Bivariate associations of covariates with past-month substance use (study 2)
eMethods. Additional details on data sources, participants, survey measures, and analysis
eReferences

This supplemental material has been provided by the authors to give readers additional information about their work.

© 2025 Vassey J et al. *JAMA Network Open.*

eFigure. Consortium diagrams for study 1 and study 2

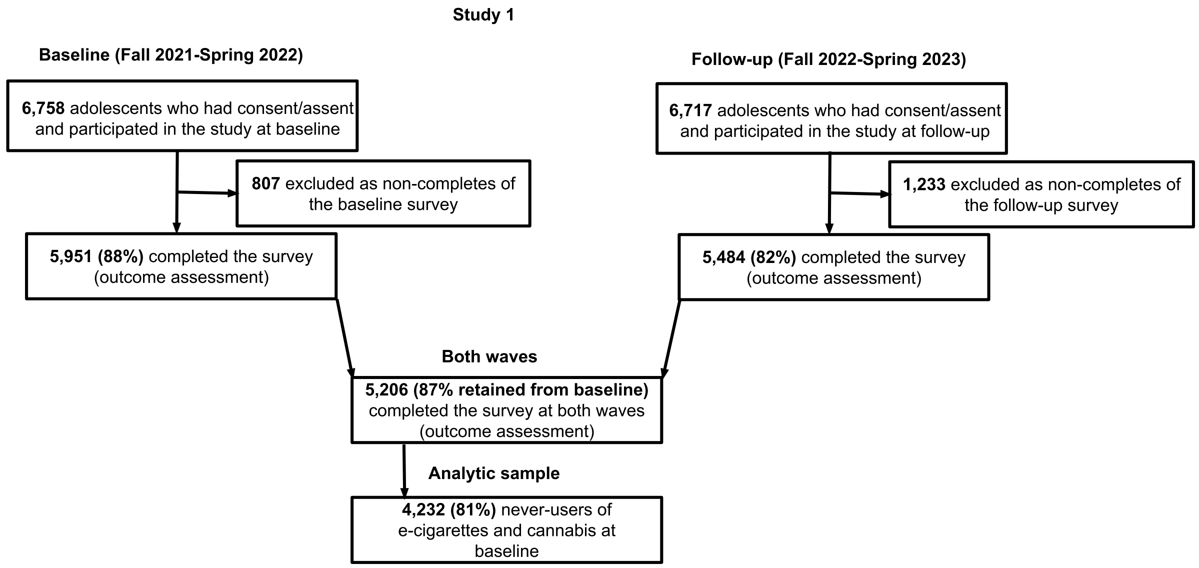

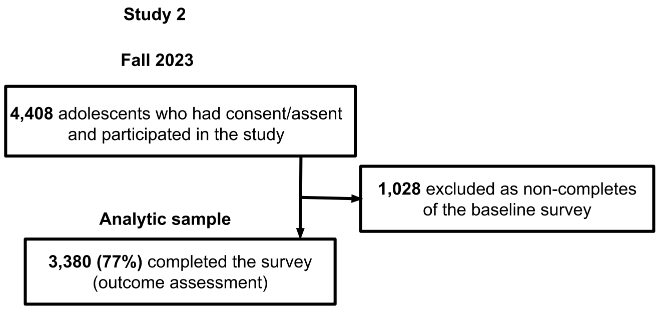

All students with parental consent and assent were eligible to participate if they were able to complete a computer-based task in English independently.

© 2025 Vassey J et al. *JAMA Network Open*.

**eTable 1. Survey participation and retention rates across two study waves (study 1)**				
**Study 1**	**Total Eligible (who had consent/assent and participated in the survey)**	**Completed survey (% of total eligible)**	**Completed both waves (% of baseline completers)**	**Retention rate (in the follow-up wave relative to baseline)**
**Cohort 1[a]**				
Baseline: Fall 2021-Spring 2022	2303	2036 (88%)	NA (baseline)	
Follow-up: Fall 2022-Spring 2023	2298	1907 (83%)	1803 (89%)	1803/2036 = 89%
**Cohort 2[b]**				
Baseline: Spring 2022	4455	3915 (88%)	NA (baseline)	
Follow-up: Spring 2023	4419	3577 (81%)	3403 (87%)	3403/3915 = 87%
**TOTAL (both cohorts)**	6758 at baseline	5951 (88%)	NA (baseline)	
	6717 at follow-up	5484 (82%)	5206 (87%)	5206/5951 = 87%

All students from 24 Los Angeles high schools with parental consent and assent were eligible to participate if they were able to complete a computer-based task in English independently.

[a] The Trends in Tobacco Use Survey (TITUS) conducted by the University of Southern California Tobacco Center of Regulatory Science (USC-TCORS).

[b] The ADVANCE survey conducted by the University of Southern California Tobacco Center of Regulatory Science (USC-TCORS).

© 2025 Vassey J et al. *JAMA Network Open*.

eTable 2. Bivariate associations of exposure to e-cigarette and cannabis posts with substance use initiation[a] (study 1)

	No. (%)[b] All (N=4232)	No. (%) Solo-e-cigarette use initiators (n=153)	No. (%) Solo-cannabis use initiators (n=166)	No. (%) Dual-use (e-cigarettes and cannabis) initiators (n=190)	No. (%) Non-users of e-cigarettes and cannabis at follow-up (n=3723)
Baseline exposure to e-cigarette posts on social media[c]					
Frequent (weekly, daily, or multiple times per day)	968 (22.9)	41 (26.8)	56[h] (33.7)	62[h] (32.6)	809[h] (21.7)
Non-frequent (monthly or less frequently)	1,415 (33.4)	55 (35.9)	47 (28.3)	66 (34.7)	1247 (33.5)
Uncertain[e]	290 (6.9)	8 (5.2)	14 (8.4)	16 (8.4)	252 (6.8)
Unexposed[f]	1102 (26.0)	27 (17.6)	23[h] (13.9)	22[h] (11.6)	1030[h] (27.7)
Unreported[g]	457 (10.8)	22 (14.4)	26 (15.7)	24 (12.6)	385 (10.3)
Baseline exposure to cannabis posts on social media[d]					
Frequent (weekly, daily, or multiple times per day)	507 (12.0)	28 (18.3)	37[h] (22.3)	43[h] (22.6)	399[h] (10.7)
Non-frequent (monthly or less frequently)	1035 (24.5)	38 (24.8)	50 (30.1)	46 (24.2)	901 (24.2)
Uncertain[e]	736 (17.4)	18 (11.8)	27 (16.3)	40 (21.1)	651 (17.5)
Unexposed[f]	1466 (34.6)	46 (30.1)	27[h] (16.3)	34[h] (17.9)	1359[h] (36.5)
Unreported[g]	488 (11.5)	23 (15.0)	25 (15.1)	27 (14.2)	413 (11.1)

Abbreviations: No. (%), Number and Percent. M (SD), Mean and Standard Deviation.

[a] Bivariate associations of exposure to e-cigarette and cannabis social media posts at baseline with four mutually-exclusive outcomes: solo-e-cigarette, solo-cannabis, dual-use initiation of e-cigarettes and cannabis, or non-initiation of e-cigarettes and cannabis at 1-year follow-up assessed among adolescent non-users of e-cigarettes and cannabis at baseline (N=4232).

[b] Percent may not add up to 100% due to rounding. Column percent is shown.

[c] Exposure to e-cigarette content on at least one of the three social media platforms: Instagram, TikTok and YouTube.

[d] Exposure to cannabis content on social media (assessed as exposure on social media in general in Cohort 1 and as exposure on at least one of the three social media platforms: Instagram, TikTok and YouTube in Cohort 2. Harmonization between Cohort 1 and Cohort 2 is described in eMethods). Instagram, TikTok and YouTube were selected since these platforms are most widely used among adolescents and host a variety of tobacco and cannabis-related image- and video-based user-generated and marketing content.

[e] *Uncertain* exposure represents responses “I don’t know” to the questions about seeing e-cigarette/cannabis posts on social media.

[f] *Unexposed* represents respondents who reported never seeing e-cigarette/cannabis posts on social media and those who were not shown the questions about exposure to e-cigarette/cannabis posts because they reported not using social media.

[g] *Unreported* (missing) responses were coded as missing and retained for the analysis.

[h] P-value < .05. *P*-values assessed for categorical variables via chi-squared tests indicate which combinations of participant characteristics by substance use status (e.g., frequently exposed to e-cigarette social media posts among cannabis use initiators) differ significantly from the expectation under the null hypothesis of no differences between the observed and the expected values.

© 2025 Vassey J et al. *JAMA Network Open*.

eTable 3. Bivariate associations of platform-specific exposure to e-cigarette posts with substance use initiation[a] (study 1)

	No. (%)[b] All (N=4232)	No. (%) E-cigarette use initiators (n=153)	No. (%) Cannabis use initiators (n=166)	No. (%) Dual-use (e-cigarettes and cannabis) initiators (n=190)	No. (%) Non-users of e-cigarettes and cannabis at follow-up (n=3723)
Baseline exposure to e-cigarette posts on TikTok					
Frequent (weekly, daily, or multiple times per day)	567 (13.4)	29 (19.0)	40[f] (24.1)	48[f] (25.3)	450[f] (12.1)
Non-frequent (monthly or less frequently)	776 (18.3)	34 (22.2)	35 (21.1)	37 (19.5)	670 (18.0)
Uncertain[c]	499 (11.8)	14 (9.2)	24 (14.5)	31 (16.3)	430 (11.5)
Unexposed[d]	1913 (45.2)	53 (34.6)	40[f] (24.1)	50[f] (26.3)	1770[f] (47.5)
Unreported[e]	477 (11.3)	23 (15.0)	27 (16.3)	24 (12.6)	403 (10.8)
Baseline exposure to e-cigarette posts on Instagram					
Frequent (weekly, daily, or multiple times per day)	590 (13.9)	23 (15.0)	39[f] (23.5)	37 (19.5)	491[f] (13.2)
Non-frequent (monthly or less frequently)	914 (21.6)	37 (24.2)	38 (22.9)	48 (25.3)	791 (21.2)
Uncertain[c]	564 (13.3)	18 (11.8)	21 (12.7)	34 (17.9)	491 (13.2)
Unexposed[d]	1682 (39.7)	52 (34.0)	39[f] (23.5)	46[f] (24.2)	1545[f] (41.5)
Unreported[e]	482 (11.4)	23 (15.0)	29 (17.5)	25 (13.2)	405[f] (10.9)
Baseline exposure to e-cigarette posts on YouTube					
Frequent (weekly, daily, or multiple times per day)	426 (10.1)	19 (12.4)	26 (15.7)	23 (12.1)	358 (9.6)
Non-frequent (monthly or less frequently)	772 (18.2)	30 (19.6)	31 (18.7)	37 (19.5)	674 (18.1)
Uncertain[c]	653 (15.4)	17 (11.1)	30 (18.1)	42 (22.1)	564 (15.1)
Unexposed[d]	1902 (44.9)	65 (42.5)	50[f] (30.1)	63[f] (33.2)	1724[f] (46.3)
Unreported[e]	479 (11.3)	22 (14.4)	29 (17.5)	25 (13.2)	403 (10.8)

Abbreviations: No. (%), Number and Percent.

[a] Bivariate association of platform-specific exposure to e-cigarette posts at baseline with four mutually-exclusive outcomes: solo-e-cigarette, solo-cannabis, dual-use initiation of e-cigarettes and cannabis, or non-initiation of e-cigarettes and cannabis at 1-year follow-up assessed among adolescent non-users of e-cigarettes and cannabis at baseline (N=4232).

[b] Percent may not add up to 100% due to rounding. Column percent is shown.

[c] *Uncertain* exposure represents responses "I don't know" to the questions about seeing e-cigarette/cannabis posts on social media.

[d] *Unexposed* represents respondents who reported never seeing e-cigarette/cannabis posts on social media and those who were not shown the questions about exposure to e-cigarette/cannabis posts because they reported not using social media.

[e] *Unreported* (missing) responses were coded as missing and retained for the analysis.

[f] P-value < .05. *P*-values assessed for categorical variables via chi-squared tests indicate which combinations of participant characteristics by substance use status (e.g., frequently exposed to e-cigarette posts on TikTok among cannabis use initiators) differ significantly from the expectation under the null-hypothesis of no differences between the observed and the expected values.

© 2025 Vassey J et al. *JAMA Network Open.*

eTable 4. Bivariate associations of covariates with substance use initiation[a] (study 1)					
	No. (%)[b] All (N=4232)	No. (%) E-cigarette use initiators (n=153)	No. (%) Cannabis use initiators (n=166)	No. (%) Dual-use (e-cigarettes and cannabis) initiators (n=190)	No. (%) Non-users of e-cigarettes and cannabis at follow-up (n=3723)
Age					
16 or younger	1139 (26.9)	39 (25.5)	27[g] (16.3)	33[g] (17.4)	1040 (27.9)
17	2766 (65.4)	98 (64.1)	127[g] (76.5)	137 (72.1)	2404[g] (64.6)
Older than 17	287 (6.8)	12 (7.8)	12 (7.2)	17 (8.9)	246 (6.6)
Unreported	40 (0.9)	4 (2.6)	0	3 (1.6)	33 (< 1)
Sex assigned at birth					
Male	1904 (45.0)	58 (37.9)	69 (41.6)	71 (37.4)	1706[g] (45.8)
Female	2204 (52.1)	90 (58.8)	88 (53.0)	109 (57.4)	1917[g] (51.5)
Undisclosed/unreported[c]	124 (2.9)	5 (3.3)	9 (5.4)	10 (5.3)	100 (2.7)
Sexual identity					
Heterosexual	3201 (75.6)	112 (73.2)	112[g] (67.5)	133 (70.0)	2844[g] (76.4)
Non-heterosexual	930 (22.0)	38 (24.8)	50[g] (30.1)	50 (26.3)	792[g] (21.3)
Unreported[c]	101 (2.4)	3 (2.0)	4 (2.4)	7 (3.7)	87 (2.3)
Ethnicity					
Hispanic/Latinx	1994 (47.1)	60 (39.2)	99[g] (59.6)	121[g] (63.7)	1714[g] (46.0)
Non-Hispanic/ Non-Latinx	2182 (51.6)	93 (60.8)	64[g] (38.6)	63[g] (33.2)	1962[g] (52.7)
Unreported[c]	54 (1.3)	0	3 (1.8)	6 (3.1)	47 (1.3)
Race					
Asian	1476 (34.9)	60 (39.2)	15[g] (9.0)	20[g] (10.5)	1362[g] (36.6)
Multi-race	831 (19.6)	43 (28.1)	42 (25.9)	34 (17.9)	712 (19.1)
Other[d]	878 (20.7)	25 (16.3)	42 (25.9)	54[g] (28.4)	757 (20.3)
White	761 (18.0)	28 (18.3)	56[g] (33.7)	47[g] (24.7)	630[g] (16.9)
Unreported[c]	286 (6.8)	2 (1.3)	7 (4.2)	18 (9.5)	178 (4.8)
Family Income					
Well off	1160 (27.4)	47 (30.7)	42 (25.3)	43 (22.6)	1028 (27.6)
Average	2335 (55.2)	84 (54.9)	79 (47.6)	104 (54.7)	2068 (55.5)
Struggling/income varies	674 (15.9)	21 (13.7)	42[g] (25.3)	36 (18.9)	575 (15.4)
Unreported[c]	63 (1.5)	1 (0.7)	3 (1.8)	7 (3.7)	52 (1.4)
Parental Education					
Below college	820 (19.4)	29 (19.0)	34 (20.5)	49 (25.8)	708 (19.0)
College or above	2919 (69.0)	111 (72.5)	121 (72.9)	127 (66.8)	2,560 (68.8)
Don't know	439 (10.4)	13 (8.5)	8 (4.8)	7[g] (3.7)	411[g] (11.0)
Unreported[c]	54 (1.3)	0	3 (1.8)	7 (3.7)	44 (1.2)
Mental Health (Internalizing disorders)[e]					
General Anxiety Disorder, M (SD)	1.02 (0.62)	1.07 (0.61)	1.12 (0.6)	1.11 (0.69)	1.01 (0.62)
Unreported[c]	166 (3.9)	8 (5.2)	4 (2.4)	8 (4.2)	146 (3.9)
Social Phobia, M (SD)	1.11 (0.76)	1.21 (0.75)	1.15 (0.81)	1.11 (0.75)	1.11 (0.75)
Unreported[c]	188 (4.4)	9 (5.9)	6 (3.6)	10 (5.3)	166 (4.5)
Other tobacco product[f] use at follow-up					
Yes	237 (6)	77[g] (50)	20[g] (12)	81[g] (43)	59[g] (2)
No	3992 (94)	76[g] (50)	146[g] (88)	109[g] (57)	3661[g] (98)
Unreported[c]	3 (<1)	0	0	0	3 (<1)
Number of friends at follow-up who used e-cigarettes or cannabis					

© 2025 Vassey J et al. *JAMA Network Open.*

At least one	1461 (34.5)	75[g] (49.0)	128[g] (77.1)	137[g] (72.1)	1121[g] (30.1)
None	2695 (63.7)	72[g] (47.1)	37[g] (22.3)	49[g] (25.8)	2537[g] (68.1)
Unreported[c]	76 (1.8)	6 (3.9)	1 (0.6)	4 (2.1)	65 (1.7)
Frequency of TikTok use					
Daily or several times per day	2296 (54.3)	98[g] (64.1)	110[g] (66.3)	13[g] (68.4)	1958[g] (52.6)
Weekly, less frequently, or no use	1482 (35.0)	34[g] (22.2)	30[g] (18.1)	36[g] (19.0)	1382[g] (37.1)
Unreported[c]	454 (10.7)	21 (13.7)	26 (15.7)	24 (12.6)	383 (10.3)
Frequency of Instagram use					
Daily or several times per day	2813 (66.5)	102 (66.7)	122[g] (73.5)	140[g] (73.7)	2449[g] (65.8)
Weekly, less frequently, or no use	959 (22.7)	29 (19.0)	17[g] (10.2)	25[g] (13.2)	888[g] (23.9)
Unreported[c]	460 (10.9)	22 (14.4)	27 (16.3)	25 (13.1)	386 (10.4)
Frequency of Youtube use					
Daily or several times per day	2754 (65.1)	88 (57.5)	97 (58.4)	97[g] (51.1)	2472[g] (66.4)
Weekly, less frequently, or no use	1027 (24.3)	44 (28.8)	44 (26.5)	69[g] (36.3)	870[g] (23.4)
Unreported[c]	451 (10.6)	21 (13.7)	25 (15.1)	24 (12.6)	381 (10.2)

Abbreviations: No. (%), Number and Percent. M (SD), Mean and Standard Deviation.

[a] Bivariate association of covariates with four mutually-exclusive outcomes: solo-e-cigarette, solo-cannabis, dual-use initiation of e-cigarettes and cannabis, or non-initiation of e-cigarettes and cannabis at 1-year follow-up assessed among adolescent non-users of e-cigarettes and cannabis at baseline (N=4232). All covariates were assessed at baseline, except for other tobacco product use and number of friends who used e-cigarettes or cannabis, which were assessed at 1-year follow-up.

[b] Percent may not add up to 100% due to rounding. Column percent is shown.

[c] Missing data on the covariates (non-response and "prefer not to respond" answers) were retained and coded as either a separate level or combined with another level for categorical variables. Missing values in the mental health continuous covariates were replaced with the corresponding mean value of that covariate.

[d] "*Other*" race category includes American Indian/Alaska Native, Black or African American, Native Hawaiian or Other Pacific Islander, and "other" race. All race categories are self-reported. They were coded as separate variables (e.g., White versus non-white).

[e] Scores range grom 0 (Never) to 3 (Always), with higher scores indicating greater severity of Generalized Anxiety Disorder symptoms based on the six-item average and Social Phobia based on the nine-item average from the Revised Children's Anxiety and Depression Scale. The specific items are listed in eMethods.

[f] Other tobacco products include combustible cigarettes, IQOS or other heated tobacco devices, oral nicotine products, big cigars, little cigars and cigarillos, and hookah.

[g] P-value < .05. P-values assessed for categorical variables via chi-squared tests or Fisher's exact tests for cell sizes 5 or fewer indicate which combinations of participant characteristics by substance use status (e.g., proportion of females among cannabis use initiators) differ significantly from the expectation under the null hypothesis of no differences between the observed and the expected values. P-values assessed via ANOVA (i.e., two mental health variables) indicate statistically significant differences in the mental health scores between substance use initiators and non-initiators.

© 2025 Vassey J et al. *JAMA Network Open.*

eTable 5. Multinomial logistic regression analysis[a] of exposure to e-cigarette and cannabis posts[b] and substance use[c] initiation (study 1)

Independent Variables[e]	Outcomes (mutually exclusive)					
	Solo e-cigarette ever-use initiation[d] (n=153)		Solo cannabis ever-use initiation[d] (n=166)		Dual ever-use (e-cigarettes and cannabis) initiation[d] (n=190)	
	AOR (95%CI)	*P* value[f]	AOR (95%CI)	*P* value[f]	AOR (95%CI)	*P* value[f]
**Model 1[g]. E-cigarette posts on social media**						
Frequent (at least weekly)	1.78 (1.03-3.10)	.16	1.82 (1.08-3.05)	.14	2.16 (1.24-3.76)	.08
Infrequent (monthly or less)	1.49 (0.90-2.48)	.15	1.12 (0.66-1.90)	.75	1.67 (0.98-2.84)	.12
Uncertain[i]	1.08 (0.46-2.55)	.87	1.84 (0.87-3.91)	.16	1.87 (0.88-4.01)	.16
Unreported[j]	1.98 (1.01-3.87)	.16	1.78 (0.93-3.38)	.14	2.00 (1.01-3.97)	.14
**Model 2[h]. Cannabis posts on social media**						
Frequent (at least weekly)	1.91 (1.07-3.39)	.08	2.44 (1.44-4.15)	.01	2.31 (1.33-4.00)	.02
Infrequent (monthly or less)	1.02 (0.62-1.69)	.93	1.83 (1.10-3.02)	.08	1.23 (0.72-2.10)	.54
Uncertain[i]	0.86 (0.44-1.66)	.70	1.57 (0.86-2.88)	.22	1.77 (0.98-3.19)	.12
Unreported[j]	1.47 (0.80-2.70)	.28	1.85 (1.00-3.43)	.12	1.78 (0.97-3.28)	.12

Abbreviations: AOR, adjusted odds ratio.

[a] Generalized Estimating Equations (GEE) Models for Multinomial Logistic Regression were applied to assess initiation of e-cigarette, cannabis and dual-use (reference: non-use of e-cigarettes and cannabis) at 1-year follow-up among adolescent never-users of e-cigarette and cannabis at baseline (N=4232). The models were adjusted for socio-demographic characteristics (age, sex assigned at birth, sexual identity, race/ethnicity, family income, parental education), mental health (internalizing disorders), other tobacco product ever-use at 1-year follow-up (combustible cigarettes, IQOS or other heated tobacco devices, oral nicotine products, big cigars, little cigars and cigarillos, and hookah), social media use habits (frequency of TikTok, Instagram and/or YouTube use), social environment around substance use (number of friends at 1-year follow-up who use e-cigarettes or cannabis), and school clustering. All covariates were assessed at baseline, except for other tobacco product use and number of friends who used e-cigarettes or cannabis, which were assessed at 1-year follow-up.

[b] Two multivariable multinomial models were conducted separately for exposure to e-cigarette and cannabis posts on social media.

[c] E-cigarette, cannabis, and dual-use initiation of e-cigarettes and cannabis were assessed. E-cigarette products include any electronic vaping device with nicotine. Cannabis products include smoking cannabis, cannabis and THC food or drinks, electronic devices to vape THC, cannabis or hash oil.

[d] Reference category for each of the three mutually-exclusive outcome categories: non-users of e-cigarettes and cannabis at follow-up (n=3727).

[e] The reference group for the independent variables (*No exposure*) combines responses of participants who reported never seeing e-cigarette/cannabis posts on social media and those who were not shown the questions about exposure to e-cigarette/cannabis posts because they reported not using social media.

[f] Adjusted p-values are reported. Benjamini-Hochberg multiple testing corrections were applied to the main predictors to control the false discovery rate at 0.05 (based on 2-tailed corrected P value).

[g] 2-Log likelihood: 4224.54 (Intercept-only), 3168.14 (Full model); Model $\chi^2(60) = 1056.40$; Nagelkerke $R^2 = 0.25$.

[h] 2-Log likelihood: 4224.54 (Intercept-only), 3162.01 (Full model); Model $\chi^2(60) = 1062.53$; Nagelkerke $R^2 = 0.25$.

[i] *Uncertain* exposure represents responses “I don’t know” to the questions about seeing e-cigarette/cannabis posts on social media.

[j] *Unreported* (missing) responses were coded as missing and retained in the data.

© 2025 Vassey J et al. *JAMA Network Open*.

eTable 6. Multinomial logistic regression analysis[a] of platform-specific exposure to e-cigarette posts[b] and substance use[c] initiation (study 1)

Independent Variables[e]	Outcomes (mutually exclusive)					
	Solo e-cigarette ever-use initiation[d] (n=153)		Solo cannabis ever-use initiation[d] (n=166)		Dual ever-use (e-cigarettes and cannabis) initiation[d] (n=190)	
	AOR (95%CI)	*P* value[f]	AOR (95%CI)	*P* value[f]	AOR (95%CI)	*P* value[f]
**Model 1[g]. E-cigarette posts on TikTok**						
Frequent (at least weekly)	1.93 (1.13-3.30)	.04	2.60 (1.62-4.17)	<.001	2.45 (1.50-4.01)	.002
Infrequent (monthly or less)	1.75 (1.02-3.01)	.07	1.69 (1.03-2.75)	.07	1.57 (0.93-2.64)	.11
Uncertain[j]	1.01 (0.51-2.01)	.98	2.01 (1.15-3.54)	.05	1.77 (1.01-3.09)	.07
Unreported[k]	1.76 (0.95-3.27)	.10	2.04 (1.16-3.60)	.05	1.65 (0.89-3.04)	.12
**Model 2[h]. E-cigarette posts on Instagram**						
Frequent (at least weekly)	1.25 (0.69-2.25)	.51	1.97 (1.22-3.17)	.06	1.54 (0.90-2.64)	.20
Infrequent (monthly or less)	1.42 (0.86-2.35)	.23	1.42 (0.89-2.27)	.22	1.61 (1.00-2.58)	.15
Uncertain[j]	1.11 (0.58-2.11)	.75	1.47 (0.81-2.66)	.24	1.80 (1.04-3.13)	.15
Unreported[k]	1.67 (0.92-3.00)	.22	1.95 (1.11-3.41)	.12	1.65 (0.91-3.02)	.22
**Model[i] 2. E-cigarette posts on YouTube**						
Frequent (at least weekly)	1.32 (0.70-2.49)	.61	1.37 (0.79-2.39)	.59	1.02 (0.56-1.88)	>.99
Infrequent (monthly or less)	1.40 (0.85-2.31)	.54	1.16 (0.73-1.84)	.72	1.02 (0.62-1.67)	>.99
Uncertain[j]	1.16 (0.65-2.10)	.74	1.53 (0.90-2.60)	.51	1.56 (0.94-2.57)	.51
Unreported[k]	1.14 (0.78-2.67)	.59	1.72 (0.99-2.99)	.63	1.32 (0.73-2.39)	.61

Abbreviations: AOR, adjusted odds ratio.

[a] Generalized Estimating Equations (GEE) Models for Multinomial Logistic Regression were applied to assess initiation of e-cigarette, cannabis and dual-use (reference: non-use of e-cigarettes and cannabis) at 1-year follow-up among adolescent never-users of e-cigarette and cannabis at baseline (N=4232). The models were adjusted for socio-demographic characteristics (age, sex assigned at birth, sexual identity, race/ethnicity, family income, parental education), mental health (internalizing disorders), other tobacco product ever-use at 1-year follow-up (combustible cigarettes, IQOS or other heated tobacco devices, oral nicotine products, big cigars, little cigars and cigarillos, and hookah), social media use habits (frequency of TikTok, Instagram and/or YouTube use), social environment around substance use (number of friends at 1-year follow-up who use e-cigarettes or cannabis), and school clustering. All covariates were assessed at baseline, except for other tobacco product use and number of friends who used e-cigarettes or cannabis, which were assessed at 1-year follow-up.

[b] Three multivariable multinomial models were conducted separately for exposure to e-cigarette posts on TikTok, Instagram and YouTube due to collinearity among exposure variables.

[c] E-cigarette, cannabis, and dual-use initiation of e-cigarettes and cannabis were assessed. E-cigarette products include any electronic vaping device with nicotine. Cannabis products include smoking cannabis, cannabis and THC food or drinks, electronic devices to vape THC, cannabis or hash oil.

[d] Reference category for each of the three mutually-exclusive outcome categories: non-users of e-cigarette and cannabis at follow-up (n=3727).

[e] Reference category for independent variables: *No exposure* that combines responses of participants who reported never seeing e-cigarette posts on TikTok, Instagram, or YouTube and those who were not shown the questions about exposure to e-cigarette posts on each of these platforms because they reported not using these platforms.

[f] Adjusted p-values are reported. Benjamini-Hochberg multiple testing corrections were applied to the main predictors to control the false discovery rate at 0.05 (based on 2-tailed corrected P value).

[g] 2-Log likelihood: 4224.54 (Intercept-only), 3154.78 (Full model); Model $\chi^2(60) = 1069.76$; Nagelkerke $R^2 = 0.25$.

[h] 2-Log likelihood: 4224.54 (Intercept-only), 3170.84 (Full model); Model $\chi^2(60) = 1053.71$; Nagelkerke $R^2 = 0.25$.

© 2025 Vassey J et al. *JAMA Network Open.*

[i] 2-Log likelihood: 4224.54 (Intercept-only), 3176.68 (Full model); Model $\chi^2(60) = 1047.87$; Nagelkerke $R^2 = 0.25$.

[j] *Uncertain* exposure represents responses “I don’t know” to the questions about seeing e-cigarette/cannabis posts on social media.

[k] *Unreported* (missing) responses were coded as missing and retained in the data.

© 2025 Vassey J et al. *JAMA Network Open*.

eTable 7. Bivariate associations of source-specific exposure to e-cigarette and cannabis posts with past-month substance use[a] (study 2)

		E-cigarette past-month use		Cannabis past-month use		Dual past-month use	
	No. (%)[b] All (N=3380)	No. (%) Use (n=170)	No. (%) Non-use (n=3201)	No. (%) Use (n=265)	No. (%) Non-use (n=3101)	No. (%) Use (n=116)	No. (%) Non-use (n=3254)
**Exposure to source-specific e-cigarette posts[c]**							
from friends							
Yes	151 (4.5)	31[e] (18.2)	120[e] (3.7)	39[e] (14.7)	112[e] (3.6)	23[e] (19.8)	128[e] (3.9)
No[d]	3229 (95.5)	139[e] (81.8)	3081[e] (96.3)	226[e] (85.3)	2989[e] (96.4)	93[e] (80.2)	3126[e] (96.1)
from micro-influencers							
Yes	195 (5.8)	25[e] (14.7)	169[e] (5.3)	44[e] (16.6)	151[e] (4.9)	22[e] (19.0)	172[e] (5.3)
No[d]	3185 (94.2)	145[e] (85.3)	3032[e] (94.7)	221[e] (83.4)	2950[e] (95.1)	94[e] (81.0)	3082[e] (94.7)
from celebrity-influencers							
Yes	131 (3.9)	16[e] (9.4)	115[e] (3.6)	23[e] (8.7)	108[e] (3.5)	14[e] (12.1)	117[e] (3.6)
No[d]	3249 (96.1)	154[e] (90.6)	3086[e] (96.4)	242[e] (91.3)	2993[e] (96.5)	102[e] (87.9)	3137[e] (96.4)
from e-cigarette brands							
Yes	407 (12.0)	32[e] (18.8)	375[e] (11.7)	42 (15.8)	365 (11.8)	25[e] (21.6)	382[e] (11.7)
No[d]	2973 (88.0)	138[e] (81.2)	2826[e] (88.3)	223 (84.2)	2736 (88.2)	91[e] (78.4)	2872[e] (88.3)
from unknown source							
Yes	611 (18.1)	28 (16.5)	583 (18.2)	42 (15.8)	569 (18.3)	18 (15.5)	593 (18.2)
No[d]	2769 (81.9)	142 (83.5)	2618 (81.8)	223 (84.2)	2532 (81.7)	98 (84.5)	2661 (81.8)
**Exposure to source-specific cannabis posts[c]**							
from friends							
Yes	161 (4.8)	37[e] (21.8)	124[e] (3.9)	59[e] (22.3)	102[e] (3.2)	26[e] (22.4)	135[e] (4.1)
No[d]	3219 (95.2)	133[e] (78.2)	3077[e] (96.1)	206[e] (77.7)	2999[e] (96.7)	90[e] (77.6)	3119[e] (95.9)
from micro-influencers							
Yes	152 (4.5)	19[e] (11.2)	133[e] (4.2)	34[e] (12.8)	118[e] (3.8)	16[e] (13.8)	136[e] (4.2)
No[d]	3228 (95.5)	151[e] (88.8)	3068[e] (95.8)	231[e] (87.2)	2983[e] (96.2)	100[e] (86.2)	3118[e] (95.8)
from celebrity-influencers							
Yes	108 (3.2)	12[e] (7.1)	96[e] (3.0)	19[e] (7.2)	89[e] (2.9)	9[e] (7.8)	99[e] (3.0)
No[d]	3272 (96.8)	158[e] (92.9)	3105[e] (97.0)	246[e] (92.8)	3012[e] (97.1)	107[e] (92.2)	3155[e] (97.0)
from cannabis brands							
Yes	243 (7.2)	17 (10.0)	226 (7.1)	30[e] (11.3)	213[e] (6.9)	15[e] (12.9)	228[e] (7.0)
No[d]	3137 (92.8)	153 (90.0)	2975 (92.9)	235[e] (88.7)	2888[e] (93.1)	101[e] (87.1)	3026[e] (93.0)
from unknown source							
Yes	353 (10.4)	19 (11.2)	333 (10.4)	36 (13.6)	317 (10.2)	15 (12.9)	337 (10.4)
No[d]	3027 (89.6)	151 (88.8)	2868 (89.6)	229 (86.4)	2784 (89.8)	101 (87.1)	2917 (89.6)

Abbreviations: No. (%), Number and Percent.

[a] Bivariate association of source-specific exposure to e-cigarette and cannabis posts with three non-mutually exclusive outcomes: past-month e-cigarette, cannabis and dual use of e-cigarettes and cannabis assessed among adolescents (N=3380). 9 observations for past-month e-cigarette use, 14 observations for past-month cannabis use and 10 observations for past-month dual-use were missing and removed from the analysis.

[b] Percent may not add up to 100% due to rounding. Column percent is shown.

[c] The source-specific exposure was assessed among those who reported at least weekly exposure to general e-cigarette/cannabis posts on social media (eMethods).

[d] Responses of those who reported never seeing e-cigarette/cannabis posts from a specific source were combined with absent responses of those who were not shown questions about the exposure to e-cigarette or cannabis posts by source (because they reported seeing non-source-specific e-cigarette or cannabis posts infrequently [monthly or less], never, or were unsure).

[e] P-value < .05. *P*-values assessed for categorical variables chi-squared tests indicated which combinations of participant characteristics by substance use status (e.g., exposure to posts from micro-influencers among past-month cannabis users) differ significantly from the expectation under the null hypothesis of no differences between the observed and the expected values.

© 2025 Vassey J et al. *JAMA Network Open.*

eTable 8. Bivariate associations of covariates with past-month substance use[a] (study 2)

		E-cigarette past-month use		Cannabis past-month use		Dual past-month use	
	**No. (%)[b] All (N=3380)**	**No. (%) Use (n=170)**	**No. (%) Non-use (n=3201)**	**No. (%) Use (n=265)**	**No. (%) Non-use (n=3101)**	**No. (%) Use (n=116)**	**No. (%) Non-use (n=3254)**
Age							
16 and younger	1079 (31.9)	42 (24.7)	1035 (32.3)	59[g] (22.3)	1016[g] (32.8)	25 (21.6)	1051 (32.3)
17	2015 (59.6)	109 (64.1)	1902 (59.4)	177 (66.8)	1830 (59)	77 (66.4)	1934 (59.4)
Older than 17	217 (6.4)	14 (8)	200 (6.2)	22 (8.3)	193 (6.2)	10 (8.6)	204 (6.3)
Unreported[c]	69 (2.0)	5 (3.0)	64 (2.0)	7 (2.6)	62 (2.0)	4 (3.4)	65 (2.0)
Sex assigned at birth							
Male	1493 (44.2)	56[g] (32.9)	1434[g] (44.8)	93[g] (35)	1391[g] (44.9)	42 (36.2)	1447 (44.5)
Female	1840 (54.4)	108[g] (63.5)	1727[g] (54.0)	166[g] (62.6)	1670[g] (53.9)	69 (59.5)	1766 (54.3)
Undisclosed/ Unreported[c]	47 (1.4)	6 (3.5)	40 (1.2)	6 (2.3)	40 (1.3)	5 (4.3)	41 (1.3)
Sexual identity							
Heterosexual	2402 (71.1)	104[g] (61.2)	2294[g] (71.7)	160[g] (60.4)	2234[g] (72.0)	71 (61.2)	2326 (71.5)
Non-heterosexual	968 (28.6)	66[g] (38.8)	898[g] (28.0)	105[g] (39.6)	858[g] (27.7)	45 (38.8)	919 (28.2)
Unreported[c]	10 (0.3)	0	9 (0.3)	0	9 (0.3)	0	9 (0.3)
Ethnicity							
Hispanic/ Latinx	1638 (48.5)	113[g] (66.5)	1519[g] (47.5)	180[g] (67.9)	1448[g] (46.7)	75[g] (64.7)	1556[g] (47.8)
Non-Hispanic/ Non-Latinx	1737 (51.4)	57[g] (33.5)	1678[g] (52.4)	85[g] (32.1)	1649[g] (53.2)	41[g] (35.3)	1694[g] (52.1)
Unreported[c]	5 (0.1)	0	4 (0.1)	0	4 (0.1)	0	4 (0.1)
Race							
Asian	1295 (38.3)	24[g] (14.1)	1270[g] (39.7)	39[g] (14.7)	1253[g] (40.4)	16[g] (13.8)	1277[g] (39.2)
Multi-race	625 (18.5)	42 (24.7)	579 (18.1)	69[g] (26.0)	552g (17.8)	26 (22.4)	596 (18.3)
Other[d]	715 (21.2)	44 (25.9)	669 (20.9)	72 (27.2)	640 (20.6)	31 (26.7)	682 (21.0)
White	640 (18.9)	54[g] (31.8)	585[g] (18.3)	78[g] (29.4)	560[g] (18.1)	39[g] (33.6)	600[g] (18.4)
Declined to respond/ Unreported[c]	105 (3.1)	6 (3.5)	98 (3.0)	7 (2.6)	96 (3.1)	4 (3.4)	99 (3.0)
Family income							
Well off	900 (26.6)	38 (22.4)	858 (26.8)	53 (20.0)	844 (27.2)	25 (21.5)	872 (26.8)
Average	1682 (49.8)	78 (45.9)	1602 (50.0)	117 (44.2)	1557 (50.2)	51 (44.0)	1628 (50.0)
Struggling	302 (8.9)	26[g] (15.3)	276[g] (8.6)	40[g] (15.1)	262[g] (8.4)	21[g] (18.1)	281[g] (8.6)
Income varies	483 (14.3)	27 (15.9)	454 (14.2)	55[g] (20.8)	426[g] (13.7)	19 (16.4)	461 (14.2)

© 2025 Vassey J et al. *JAMA Network Open*.

Unreported[c]	13 (0.4)	1 (0.5)	11 (0.3)	0	12 (0.4)	0	12 (0.4)
Parental education							
Below college	745 (22.0)	40 (23.5)	704 (22.0)	66 (24.9)	677 (21.8)	24 (20.7)	719 (22.0)
College or above	2437 (72.1)	121 (71.2)	2310 (72.2)	188 (70.9)	2239 (72.2)	85 (73.3)	2346 (72.1)
Don't know	193 (5.7)	9 (5.3)	182 (5.7)	11 (4.2)	180 (5.8)	7 (6.0)	184 (5.7)
Unreported[c]	5 (0.2)	0	5 (0.1)	0	5 (0.2)	0	5 (0.2)
Mental Health (internalizing disorders)[e]							
General Anxiety Disorder, M (SD)	0.98 (0.73)	1.18[g] (0.75)	0.97[g] (0.69)	1.13[g] (0.7)	0.97[g] (0.7)	1.18[g] (0.73)	0.98[g] (0.7)
Unreported[c]	325 (9.6)	45 (26.5)	292 (9.1)	52 (19.6)	296 (9.5)	32 (27.6)	307 (9.4)
Social Phobia, M (SD)	1.27 (0.78)	1.42[g] (0.72)	1.26[g] (0.74)	1.36[g] (0.72)	1.26[g] (0.74)	1.45[g] (0.71)	1.26[g] (0.74)
Unreported[c]	336 (9.9)	50 (29.4)	298 (9.3)	53 (20.0)	305 (9.8)	33 (28.4)	317 (9.7)
Other tobacco product[f] use in the past six months							
Yes	181 (5.4)	74[g] (43.5)	101[g] (3.2)	84[g] (31.7)	87[g] (2.8)	58[g] (50.0)	117[g] (3.6)
No	3199 (94.6)	96[g] (56.5)	3100[g] (96.8)	181[g] (68.3)	3014[g] (97.2)	58[g] (50.0)	3137[g] (96.4)
Number of friends who used e-cigarettes or cannabis							
At least one	1126 (33.3)	148[g] (87.0)	976[g] (30.5)	224[g] (84.5)	900[g] (29.0)	105[g] (90.5)	1019[g] (31.3)
None	2146 (63.5)	12[g] (7.1)	2133[g] (66.6)	31[g] (11.7)	2114[g] (68.2)	5[g] (4.3)	2141[g] (65.8)
Unreported[c]	108 (3.2)	10 (5.9)	92 (2.9)	10 (3.8)	87 (2.8)	6 (5.2)	94 (2.9)
Frequency of TikTok, Instagram and/or YouTube use							
Daily or several times per day	2609 (77.2)	129 (75.9)	2478 (77.4)	210 (79.2)	2396 (77.3)	87 (75.0)	2521 (77.5)
Weekly, monthly or less frequently, or none	484 (14.3)	5[g] (2.9)	479[g] (15.0)	8[g] (3.0)	476[g] (15.3)	4[g] (3.4)	480[g] (14.7)
Unreported[c]	287 (8.5)	36 (21.2)	244 (7.6)	47 (17.8)	229 (7.4)	25 (21.6)	253 (7.8)

Abbreviations: No. (%), Number and Percent. M (SD), Mean and Standard Deviation.

[a] Bivariate associations of covariates with three non-mutually exclusive outcomes: past-month e-cigarette, cannabis, and dual use of e-cigarettes and cannabis assessed among adolescents (N=3380). 9 observations for past-month e-cigarette use, 14 observations for past-month cannabis use and 10 observations for past-month dual-use were missing and removed grom the analysis.

[b] Percent may not add up to 100% due to rounding. Column percent is shown.

[c] Missing data on the covariates (non-response and "prefer not to respond" answers) were retained and coded as either a separate level or combined with another level for categorical variables. Missing values in the mental health continuous covariates were replaced with the corresponding mean value of that covariate.

[d] "*Other*" race category includes American Indian/Alaska Native, Black or Agrican American, Native Hawaiian or Other Pacific Islander, and "other" race. All race categories are self-reported. They were coded as separate variables (e.g., White versus non-white).

[e] Scores range grom 0 (Never) to 3 (Always), with higher scores indicating greater severity of Generalized Anxiety Disorder symptoms based on the six-item average and Social Phobia based on the nine-item average from the Revised Children's Anxiety and Depression Scale. The specific items are listed in eMethods.

[f] Other tobacco products include combustible cigarettes, IQOS or other heated tobacco devices, oral nicotine products, big cigars, little cigars and cigarillos, and hookah.

[g] P-value < .05. *P*-values assessed for categorical variables via chi-squared tests indicate which combinations of participant characteristics by substance use status (e.g., exposure to posts grom micro-influencers among past-month cannabis users) digger significantly grom the expectation under the null-hypothesis of no differences between the observed and the expected values. P-values assessed via ANOVA (i.e., two mental health variables) indicate statistically significant differences in the mental health scores between past-month substance users and non-users.

© 2025 Vassey J et al. *JAMA Network Open.*

eMethods. Additional details on data sources, participants, survey measures, and analysis

Background. In this study, we analyzed the Trends in Tobacco Use Survey (TITUS) survey and the ADVANCE survey conducted by the University of Southern California Tobacco Center of Regulatory Science (USC-TCORS). TITUS, referred to as Cohort 1 in Study 1, is a longitudinal annual survey that started in 2019 and was designed to gather data on tobacco (including e-cigarette) use behaviors, attitudes toward and perceptions of tobacco products and health risks from tobacco use among adolescents in 9th to 12th grades from 13 Los Angeles high schools. ADVANCE, referred to as Cohort 2 in Study 1, is a longitudinal semi-annual survey that started in 2020 with the goal of better understanding the health and well-being of adolescents in 9th to 12th grades from 11 Los Angeles high schools. Study 1 included Wave 3/Wave 4 of TITUS (Cohort 1) and Wave 4/Wave 6 of ADVANCE (Cohort 2). For Study 1, data from TITUS Wave 3 and ADVANCE Wave 4, collected over Fall 2021-Spring 2022, were merged and analyzed as baseline, while merged data from TITUS Wave 4 and ADVANCE Wave 6, collected over Fall 2022-Spring 2023, were analyzed as a 1-year follow-up. Study 2, ADVANCE, included data from Wave 7, collected over Fall 2023.

Participants and sampling. Approximately 70 public high schools in Southern California were approached about participating in this study. These schools were chosen because of their diverse demographic characteristics, including a balance of race/ethnicity, rural/urban, and socioeconomic status to approximate the general demographic of Southern California youth. 24 schools that agreed to participate in TITUS and ADVANCE surveys are based in Los Angeles, San Bernadino, Riverside, Orange, and Imperial County. All students with parental consent and assent were eligible to participate if they were able to complete a computer-based task in English independently.

Procedures. Students whose parents submitted a consent form received a $5 gift card whether the consent decision was “yes” or “no.” During in-class data collection, students received a small trinket (e.g., candy, stickers). Among all the 9290 contacted parents for both cohorts combined, 2532 (27%), which is 1778 parents for the ADVANCE survey and 754 parents for the TITUS survey, never submitted a consent decision for their child.

Information about survey procedures during COVID-19. For TITUS (Cohort 1), students were originally enrolled prior to COVID (in-school recruitment). However, school shutdowns in March 2020 halted TITUS recruitment for the remainder of Wave 1. Therefore, an additional 3 schools were recruited in Wave 2. That year, data collection occurred remotely during synchronous online classes. In Wave 3 and Wave 4, data collection resumed in-class/in-person. For ADVANCE (Cohort 2), schools and participants were enrolled during the remote and hybrid learning phases of California’s COVID-19 response. However, the pandemic likely had minimal to no negative impact on student recruitment. Students were invited to participate either during in-person classes or through remote/online classes, depending on the school’s teaching modality. A USC researcher was present and engaged with students regardless of the learning format. For students who were absent during the scheduled survey, the opportunity to complete it on their own time was offered, with a $10 gift card incentive. For both ADVANCE and TITUS cohorts, significant effort was made to engage chronically absent students, including calls and emails to students, incentivizing teachers to provide survey reminders, and sharing key study information via Google Classroom.

Survey items related to outcomes and predictors, and harmonization of variables in Cohort 1 and Cohort 2, in Study 1.

Harmonization was required for variables where survey items in Cohort 1 and Cohort 2 in Study 1 varied due to differences in survey design by two independent study teams.

E-Cigarette Initiation

Survey question, Cohort 1: *“Have you ever used the following substances in your life?”* with *yes/no* response options for each of the following products:

1) *e-cigarettes with nicotine (E-cigs, personal vaporizer)*
2) *JUUL*
3) *disposable e-cigarette devices (Puff Bar, Mojo, Cali Bar, Hyppe Bar, Ezzy, etc.)*
4) *other e-cigarette products*

Survey question, Cohort 2: *"Have you ever used an electronic cigarette for vaping nicotine?"* with a *yes/no* response option.

© 2025 Vassey J et al. *JAMA Network Open*.

Harmonization: Responses in Cohort 1 were combined into one binary response: *yes* (if at least one product was used) vs. *no* to align with Cohort 2. The harmonized variable indicates whether any e-cigarette product was ever used.

Cannabis Use Initiation

Survey questions, Cohort 1 and Cohort 2: *"Have you ever used the following substances in your life?"* with *yes/no* response options for each of the following products:

1) Smoking marijuana (pot, weed, hash, reefer, bud, or grass)
2) Marijuana or THC foods or drinks (pot brownies, edibles, cookies, cakes, butter, oil)
3) Electronic device to vape THC or hash oil (liquid pot, marijuana oil, weed pen, PAX Era)

Responses were combined into one binary response as *yes* (if at least one product was used) vs. *no*. The outcome variable indicates whether any cannabis product was ever used.

"Cannabis" and "marijuana" terms were used in the survey questionnaires interchangeably.

Dual-Use Initiation

Initiation of both e-cigarettes and cannabis (*yes* responses to both binary-coded e-cigarette and cannabis use initiation outcomes) was considered dual-use initiation.

Exposure to E-Cigarette Posts

Survey questions, Cohort 1 and Cohort 2: "*How often do you see posts about e-cigarette products on the following social media?" Instagram, TikTok, and YouTube* (assessed separately per each platform), with response options including:

Several times per day, daily, weekly, monthly or less, never, don't know.

Exposure was analyzed separately for each of the three platforms and as a general exposure to e-cigarette posts on social media by combining exposure on any of the three platforms - TikTok, Instagram, and/or YouTube - into a single variable.

Responses for general and platform-specific exposures were dichotomized to frequent exposure (*several times per day, daily, weekly*) versus all the other response choices, including unreported responses retained in the analysis, combined. The exposures were also analyzed more granularly: frequent (*several times per day, daily, weekly*), infrequent (*monthly or less, never*), uncertain (*don't know*), unreported (missing values retained in the analysis), and non-exposure (*never*). In the analysis, non-exposure combined responses of participants who reported never seeing e-cigarette posts on Instagram, TikTok and/or YouTube and those who were not shown the questions about exposure to e-cigarette posts because they reported not using these social media platforms.

Exposure to Cannabis Posts

Survey question, Cohort 1: *"How often do you see posts about cannabis products on any social media platform?"* with response options including:

Several times per day, daily, weekly, monthly or less, never, don't know.

Survey question, Cohort 2: "*How often do you see posts about cannabis products on the following social media?" Instagram, TikTok, and YouTube* (assessed separately per each platform), with response options including:

Several times per day, daily, weekly, monthly or less, never, don't know.

© 2025 Vassey J et al. *JAMA Network Open*.

Harmonization: Responses from Cohort 2 were combined into a single variable (exposure on any of the three platforms [TikTok, Instagram, YouTube]) to match Cohort 1. Instagram, TikTok and YouTube were selected since these platforms are most widely used among adolescents and host a variety of tobacco or cannabis-related image- and video-based user-generated and marketing content.

Responses were dichotomized to frequent exposure (*several times per day, daily, weekly*) versus all the other response choices, including unreported responses retained in the analysis, combined. The exposure was also analyzed more granularly: frequent (*several times per day, daily, weekly*), infrequent (*monthly or less, never*), uncertain (*don't know*), unreported (missing values retained in the analysis), and non-exposure (*never*). In the analysis, non-exposure combined responses of participants who reported never seeing cannabis posts on social media and those who were not shown the questions about exposure to cannabis posts because they reported not using social media.

Survey items related to outcomes and predictors in Study 2

Past-Month E-Cigarette Use

Survey question: *"In the last 30 days, how many total days have you used an electronic cigarette for vaping nicotine?"* with response option including:

0 days, 1-2 days, 3-5 days, 6-9 days, 10-19 days, 20-29 days, All 30 days

Due to low cell counts for granular use (1–2 days: 2%, 3+ days: 3%), responses were dichotomized as any use (≥1 day) versus no use (0 days).

Past-month Cannabis Use

Survey question: *"In the last 30 days, how many total days have you used the following substances?"* with response options including:

0 days, 1-2 days, 3-5 days, 6-9 days, 10-19 days, 20-29 days, All 30 days

1) Smoking marijuana (pot, weed, hash, reefer, bud, or grass)
2) Marijuana or THC foods or drinks (pot brownies, edibles, cookies, cakes, butter, oil)
3) Electronic device to vape THC or hash oil (liquid pot, marijuana oil, weed pen, PAX Era)

Due to low cell counts for granular use (1–2 days: 3%, 3+ days: 5%), responses were dichotomized as any use (≥1 day) of any cannabis product versus no use (0 days) of all the products.

Past-Month Dual Use

Past-month use of both e-cigarettes and cannabis (≥1 day use of both binary-coded e-cigarettes and cannabis) was considered past-month dual-use.

Exposure to E-Cigarette Posts from a Specific Source

If respondents selected response choices *Several times per day, daily, or weekly* to the question: "*How often do you see posts about e-cigarette products?"* on either Instagram, TikTok, or YouTube, they were shown the following question:

"Who posted it?" [referring to e-cigarette content that respondents' saw] with response choices: a) *friend;* b) *celebrity;* c) *micro-influencer*; d) *any e-cigarette company, brand or store;* e) *don't know* with *yes/no* response options.

Survey instrument contained a prompt that defined micro-influencers as models and/or brand ambassadors who have 1,000 or more social media followers and are regarded as experts in certain topics.

© 2025 Vassey J et al. *JAMA Network Open*.

Responses were dichotomized as *yes/no* exposure by source. Responses of those who reported no exposure were combined with responses of those for whom source-specific exposure was not assessed. The latter group remained in the analysis and included respondents who answered *monthly or less*, *never*, *don't know*, and unreported, to the question about exposure to general e-cigarette-related posts: *"How often do you see posts about e-cigarette products?"* on either Instagram, TikTok, or YouTube.

Exposure to Cannabis Posts from a Specific Source

If respondents selected response choices *Several times per day, daily, or weekly* to the question "*How often do you see posts about cannabis products?"* on either Instagram, TikTok, or YouTube, they were shown the following question:

"Who posted it?" [referring to cannabis content that respondents' saw] with response choices: a) *friend;* b) *celebrity;* c) *micro-influencer*; d) *a cannabis company, brand or store;* e) *don't know* with *yes/no* response options.

Survey instrument contained a prompt that defined micro-influencers as models and/or brand ambassadors who have 1,000 or more social media followers and are regarded as experts in certain topics.

Responses for each source were dichotomized as *yes/no* exposure. Responses of those who reported no exposure were combined with responses of those for whom source-specific exposure was not assessed. The latter group remained in the analysis and included respondents who answered *monthly or less*, *never*, *don't know*, and unreported, to the question about exposure to general cannabis-related posts: *"How often do you see posts about cannabis products?"* on either Instagram, TikTok, or YouTube.

Covariates (self-reported characteristics) for Study 1 and Study 2

Age

Survey question:

"How old are you?" (in years)

Age was coded as a categorical variable with the following brackets: 16 and younger, 17, older than 17.

Students also answered the following questions: "*What month were you born?" "What year were you born?"* Responses to the *"How old are you?"* question were validated against these questions.

Sex Assigned at Birth

Survey question: "*What is your sex assigned at birth?"* with *Male/Female/Prefer not to disclose* response choices.

Sexual Identity

Survey question: "*Do you consider yourself to be"* with the following response choices: *Straight/Heterosexual/Asexual/Bisexual/Gay/Lesbian/ Pansexual/Queer/Questioning or unsure/Another identity not listed here (specify)/Prefer not to disclose*

The covariate was coded as a binary variable: *Straight/Heterosexual vs. all the other categories.*

Ethnicity

Survey question: "*Are you Hispanic, Latino, Latina, or Latinx?"* with *yes/no* response choices.

Race

Survey question: "*Which of the following races do you identify with?"* with the following response choices: *American Indian or Alaska Native/Asian/Black or African American/Native Hawaiian or Pacific Islander/White/Multi-ethnic or Multi-racial/Another race/racial identity not listed here/Decline to respond/prefer not to disclose*

American Indian/Alaska Native, Black or African American, Native Hawaiian or Other Pacific Islander were grouped and coded as "other" race. All race groups were coded as separate variables (e.g., White versus non-white).

Family Income[1]

Survey question: "*Think about your family when you were growing up, from birth to age 16. Would you say your family during that time was…":*

Pretty well off financially/About average/Financially struggling or in poverty/It varied

Parental Education

Survey question: "*What is the highest grade completed by either parent or guardian?"* with the following response choices: *8th grade or less/Some high school/High school graduate/Some college/College graduate/Advanced degree/I don't know*

The covariate was coded as a categorical variable: *College or above/Below college/Don't know.*

Mental Health (Internalizing Disorders)[2]

The Revised Children's Anxiety and Depression Scale to assess Generalized Anxiety Disorder (GAD; 6 items) and Social Phobia (9 items) was used.[2] Each item was rated on a 4-point scale: "Never" (0), "Sometimes" (1), "Often" (2), and "Always" (3), with higher scores indicating greater symptom severity. Composite mean scores were calculated for each scale (GAD: Cronbach's α = .90; Social Phobia: α = .92). These continuous composite scores were included as covariates in the analysis.

Survey prompt: *Next we will be asking questions about how you have been feeling. Please be open and honest in your responding and answer how you feel is most appropriate in describing you,* with the following response choices:

Generalized Anxiety Disorder (GAD):

I worry about things
I worry that something awful will happen to someone in my family
I worry that bad things will happen to me
I think about death
I worry about what is going to happen
I worry that something bad will happen to me

Social Phobia:

I worry when I think I have done poorly at something
I feel scared when I have to take a test
I feel worried when I think someone is angry with me
I worry that I will do badly at my schoolwork
I worry I might look foolish
I worry about making mistakes
I worry what other people think of me
I feel afraid if I have to talk in front of my class
I feel afraid that I will make a fool of myself in front of people

Other Tobacco Product Use

© 2025 Vassey J et al. *JAMA Network Open.*

Survey questions: Study 1: *"Have you ever used the following substances in your life?"* and Study 2: *"Have you ever used the following substances in the past six months?"* with *yes/no* response options for each of the following products:

A few puffs of a cigarette (Marlboro, Camel, Newport)/A whole cigarette (Marlboro, Camel, Newport)/IQOS or other heated tobacco devices (devices that heat, but don't burn, shredded tobacco leaves)/Dissolvable tobacco like Snus (moist powder smokeless tobacco, Swedish snus)/Nicotine pouches (smokeless nicotine pouches, Zyn, Velo, On!)/Any flavored nicotine gum, lozenges, tablets, and/or gummies (Lucy, Rogue, Velo, Zyn, On!, Krave)/Big cigars (stogies, cubans)/Little cigars or cigarillos (clippers, swisher sweets, black and milds)/Hookah water pipe (narghile, shisha)

Responses were combined into a one binary response: *yes* (if at least one product was used) vs. *no.*

Number of Friends Who Used E-cigarettes or Cannabis

Survey question: *"How many of your FIVE (5) closest friends use any of these substances*?" with *yes/no* response choice for each of the following e-cigarette and cannabis product:

Study 1, Cohort 1:
1) *e-cigarettes with nicotine (E-cigs, personal vaporizer)*
2) *JUUL*
3) *disposable e-cigarette devices (Puff Bar, Mojo, Cali Bar, Hyppe Bar, Ezzy, etc.)*
4) *other e-cigarette products*

Study 1, Cohort 2: *Electronic cigarettes for vaping nicotine (E-cigs, vaporizer, JUUL, Puff Bar)*

Harmonization: Responses in Cohort 1 were combined into one binary response: *yes* (if at least one product was used) vs. *no* to align with Cohort 2. The harmonized variable indicates number of friends who use any e-cigarette product.

Study 1, Cohort 1 and Cohort 2:
1) Smoking marijuana (pot, weed, hash, reefer, bud, or grass)
2) Marijuana or THC foods or drinks (pot brownies, edibles, cookies, cakes, butter, oil)
3) Electronic device to vape THC or hash oil (liquid pot, marijuana oil, weed pen, PAX Era)

Study 2
1) Electronic cigarettes for vaping nicotine (E-cigs, vaporizer, JUUL, Puff Bar)
2) Smoking marijuana (pot, weed, hash, reefer, bud, or grass)
3) Marijuana or THC foods or drinks (pot brownies, edibles, cookies, cakes, butter, oil)
4) Electronic device to vape THC or hash oil (liquid pot, marijuana oil, weed pen, PAX Era)

The covariate was coded as a binary variable: any number of friends above 0 using e-cigarettes and/or any cannabis product vs. no friends using any of these products.

Frequency of TikTok/Instagram/YouTube Use

Survey question: *"How often do you visit the following social media sites?"* with the following response choices for TikTok, Instagram or YouTube assessed separately: *I do not use this social media platform/Monthly or less/Weekly/Daily/Several times per day/Don't know*

The covariate was coded as a binary variable: several times a day or daily use of TikTok, Instagram and/or YouTube (indicating frequent use) vs the other response choices.

All the measures, except for family income and mental health, were adapted from Population Assessment of Tobacco and Health [PATH] Study.[3]

© 2025 Vassey J et al. *JAMA Network Open*.

eReferences

1. Understanding America Study. Accessed March 6, 2025. https://uasdata.usc.edu/index.php

2. Ebesutani C, Bernstein A, Nakamura BJ, Chorpita BF, Weisz JR. A Psychometric Analysis of the Revised Child Anxiety and Depression Scale—Parent Version in a Clinical Sample. *J Abnorm Child Psychol*. 2010;38(2):249-260. doi:10.1007/s10802-009-9363-8

3. PATH (Population Assessment of Tobacco and Health) Study - Home. Accessed May 10, 2024. https://pathstudyinfo.nih.gov/

© 2025 Vassey J et al. *JAMA Network Open*.
