# Supplementary material for: E-Cigarette and Cannabis Social Media Posts and Adolescent Substance Use
Source: JAMA Netw Open. 2025 Jun 24;8(6):e2517611. doi: 10.1001/jamanetworkopen.2025.17611 (PMC12188343; doi:10.1001/jamanetworkopen.2025.17611)
Supplement: Supplement 2. — Data Sharing Statement [file jamanetwopen-e2517611-s002.pdf]

Data Sharing Statement

Vassey. E-Cigarette and Cannabis Social Media Posts and Adolescent Substance Use. *JAMA Netw Open*. Published June 24, 2025. doi:10.1001/jamanetworkopen.2025.17611

Data
Data available: Yes
Data types: Data dictionary, Deidentified participant data
How to access data: Data will be made available upon request that can be emailed to vassey@usc.edu
When available: With publication

Supporting Documents
Document types: Statistical/analytic code
How to access documents: Data will be made available upon request that can be emailed to vassey@usc.edu
When available: With publication

Additional Information
Who can access the data: researchers requesting the data
Types of analyses: researchers requesting the data for any purposes
Mechanisms of data availability: with a signed data access agreement
